# A comprehensive longitudinal analysis of changes during Attention Deficit Hyperactive Disorder pharmacological treatments: Relationships between clinical measures, QbCheck and Conners CPT‐II

**DOI:** 10.1002/jcv2.70021

**Published:** 2025-06-20

**Authors:** Seungjae Lee, Renee Testa, Beth Johnson, David Coghill

**Affiliations:** ^1^ Turner Institute for Brain and Mental Health Monash University Clayton Victoria Australia; ^2^ Department of Paediatrics Faculty of Medicine, Dentistry and Health Sciences University of Melbourne Melbourne Victoria Australia; ^3^ Murdoch Children's Research Institute Royal Children's Hospital Parkville Victoria Australia

**Keywords:** ADHD, clinical outcomes, cognition, Conners CPT‐II, measurement‐based care, pharmacological treatment, QbCheck

## Abstract

**Background:**

This study aims to evaluate the clinical value of using QbCheck in routine Attention Deficit Hyperactive Disorder (ADHD) clinics by investigating longitudinal inter‐domain relationships between objective neurocognitive outcomes of QbCheck and subjective clinical outcomes: ADHD core symptoms, impairment and quality of life (QoL).

**Method:**

The study was conducted alongside the participants' standard pharmacological treatment for their ADHD. Thirty‐four clinically diagnosed participants with ADHD completed baseline and follow‐up neurocognitive assessments. Bayesian paired *t*‐tests and bivariate correlations were used to examine changes over time and relationships between key variables.

**Results:**

Bayesian analyses showed correlations between QbCheck and continuous performance test (CPT)‐II for the same constructs, except for omission errors. In the test‐retest results of QbCheck, measures remained stable, though moderate evidence supported changes in reaction time variability (BF_10_ = 5.57) and anecdotal evidence for commission errors (BF_10_ = 1.72). In measuring treatment effect, there was moderate to extreme evidence for reductions in self‐ and informant‐rated ADHD symptoms and clinician‐rated impairment severity. In contrast, informant‐rated QoL showed weak evidence for a difference (BF_10_ = 1.81), and self‐rated QoL showed no change (BF_10_ = 0.67). QbCheck showed moderate to extreme evidence for most changes, with weak evidence for reaction time (BF_10_ = 1.38), while CPT‐II showed strong evidence for commission errors, weak evidence for response time variability, and no evidence for omission errors or reaction time. Weak evidence suggested moderate associations between QbCheck MicroEvent X and informant‐rated ADHD symptom severity (BF_10_ = 2.40, *r* = 0.47) and CPT‐II commission errors and self‐rated ADHD symptom severity (BF_10_ = 2.44, *r* = 0.39).

**Conclusion:**

QbCheck is a valid tool for assessing neurocognitive changes in ADHD treatment but should not replace clinical outcome measures or be used as a proxy for behavioural assessments. Caution is needed when relying on a single outcome measure, emphasizing the need for multi‐source assessments to capture the full impact of treatment.

**Key points**

- QbCheck is a valid tool for assessing certain neurocognitive changes during Attention Deficit Hyperactive Disorder (ADHD) pharmacological interventions.
- Low inter‐rater reliability in clinical outcomes highlights the need for objective measures to supplement.
- Changes in QbCheck movement scores (MicroEventX) align with informant‐rated ADHD total symptom severity, offering a useful alternative when multi‐source clinical assessments are difficult.
- Change in continuous performance test (CPT)‐II commission error correlated with changes in self‐rated ADHD total symptom severity.
- The weak or absent correlation between CPTs and clinical outcomes suggests that these measures assess different aspects of treatment effects. This highlights the limitation of relying on a single outcome measure to capture the full effects of ADHD treatment.
- Future research should investigate the long‐term impact of neurocognitive changes on academic performance, occupational success, and social interactions over extended follow‐up periods.

## INTRODUCTION

Attention Deficit Hyperactive Disorder (ADHD) treatment guidelines from various countries advocate for measurement‐based care (MBC), emphasising regular assessments of clinical outcomes, including core symptoms, functional impairments, and quality of life (QoL) for the ongoing evaluation of treatment effectiveness (Australasian ADHD Professionals Association [AADPA], 2022; CADDRA – Canadian ADHD Resource Alliance, 2020; National Institute for Health and Care Excellence [NICE], 2018). However, current ADHD clinical outcome measures have been criticised for their sole reliance on subjective reports and lack of objectivity (Weiss & Stein, 2022). Some of subjective reports limitations include vulnerability to bias (Emser et al., 2018; Söderström et al., 2014), prone to being influenced by context (e.g., social/parental expectations, social desirability, co‐occurring conditions; Alarachi et al., 2024; Hareendran et al., 2015; Mulraney et al., 2022), children and adolescents may struggle to accurately report their own symptoms (Coxe et al., 2021; Peterson et al., 2024; Wilens et al., 2006), informant reports may not fully capture subtle differences in experiences (Garcia‐Rosales et al., 2024), low interrater reliability, and high variability in the interpretation of questionnaires (Bédard et al., 2015; Bussing et al., 2008; Butzbach et al., 2021; Garcia‐Rosales et al., 2024; Slobodin & Davidovitch, 2019; Weiss & Stein, 2022; Wilens et al., 2006). Alongside these concerns, practical challenges also arise, as MBC can be time‐consuming and costly, making it difficult to implement regularly in clinical practice (Hall, Selby, et al., 2016; Higdon et al., 2020; Martin‐Cook et al., 2021). Previous studies (Emser et al., 2018; Evans et al., 2021; Hall, Taylor, et al., 2016; Hall, Valentine, et al., 2016; Lee et al., 2022; Rapport, et al., 2000) have suggested that continuous performance tasks/tests (CPTs) may serve as a complementary objective measure to assist MBC.

QbCheck is a CPT designed to provide objective data on neurocognitive functions, including inattention, impulsivity, and hyperactivity (Hult et al., 2018; Ulberstad et al., 2020). In Australia, QbCheck is the first CPT approved by the Therapeutic Goods Administration for clinical use. However, before the current study, there was limited psychometric data on QbCheck's reliability and validity in assessing neurocognitive functions, with none of the existing research including an Australian sample. Ulberstad et al. (2020) examined the psychometric properties of QbCheck using data from 69 individuals with ADHD and 73 controls without ADHD, aged 12–60, in Germany and Sweden. Their findings demonstrated high test‐retest reliability, with intra‐class correlation coefficients ranging from 0.82 to 0.96. While the study reported strong validity, the QbCheck data were compared only to the earlier version of QbTest from the same company. Establishing convergent validity by comparing QbCheck to other widely used CPTs, such as Conners CPT‐II, would provide more meaningful insights. Conners CPT‐II is a well‐established tool for measuring cognitive function in research (Lichtenstein et al., 2019). Previous studies (Egeland & Kovalik‐Gran, 2010; Pagán et al., 2023; Varela et al., 2024) highlight the CPT‐II's moderate test‐retest reliability, reasonable construct validity for attention and impulsivity, and sensitivity to changes in response to medication. However, it has limited specificity, struggling to distinguish ADHD from typical behaviours and comorbid conditions, as well as limited generalizability.

ADHD pharmacological treatments, particularly stimulant medications such as methylphenidate and amphetamines, have been shown to improve multiple neurocognitive functions, including sustained attention, short‐term memory, working memory, and response inhibition, but not exclusively (Lee et al., 2022). For example, CPT results consistently show significant improvements in reaction time consistency and reductions in errors after ADHD medication initiation (Cortese et al., 2015; Tamm et al., 2019). Despite their potential to measure changes, studies have shown that CPTs results have limited value as proxies for subjective symptom ratings (Evans et al., 2021; Lee et al., 2022). A larger‐scale Swedish cross‐sectional study (Tallberg et al., 2019), including 118 diagnostic and 186 medication titration cases, found weak correlations between Conners CPT‐II and Swanson, Nolan and Pelham (SNAP‐IV) Scale parent (*r* = 0.17, *p* = 0.11) and teacher (*r* = 0.15, *p* = 0.15) ratings. These cross‐sectional studies have primarily assessed the CPT's diagnostic value, but their potential for tracking treatment outcomes over time remains largely unexplored (Cortese et al., 2015; Lee et al., 2022; Sonuga‐Barke et al., 2013; Ulberstad et al., 2020).

Recent longitudinal research has attempted to explore the potential of CPTs in monitoring ADHD treatment outcomes. Cedergren et al. (2022) conducted a single‐centre prospective study assessing ADHD symptoms (ADHD‐Rating Scale [ADHD‐RS]) and neurocognitive functions (QbTest [the predecessor of QbCheck]) at baseline, 1 month, and 12 months after the treatment. The result showed significant symptom reduction and improvement of QbTest results after 12 months of pharmacological treatments. Weak but significant correlations between ADHD‐RS hyperactivity and QbTest impulsivity were reported at 1 month. However, no significant long‐term correlations were found between QbTest and ADHD‐RS outcomes at 12 months. These findings highlight the potential benefits of monitoring neurocognitive function with CPTs during ADHD treatment but suggest limited usefulness in predicting or serving as proxies for ADHD symptom changes. Despite these advancements, the broader clinical utility of CPTs remains poorly understood. Specifically, there is a lack of comprehensive investigation into how CPT results may align with broader clinical outcomes, such as QoL and functional impairment (Coghill et al., 2019), beyond ADHD core symptoms. Understanding these relationships is key to leveraging CPTs as objective measures for MBC. This study aims to fill knowledge gaps by examining longitudinal associations between QbCheck neurocognitive results and broader clinical outcome measures of ADHD during pharmacological treatment.

The study objectives include (1) evaluating whether the QbCheck can be used to measure changes during ADHD pharmacological treatment and (2) exploring the correlations between longitudinal changes in QbCheck results and three domains of clinical outcomes: ADHD core symptoms, impairment, and quality of life (QoL). Hypotheses to answer these research questions are as follows:

1. QbCheck and CPT‐II results will improve after pharmacological interventions.
2. Changes in QbCheck results will be correlated with changes in Conners CPT‐II results.
3. Changes in QbCheck and CPT‐II results will not be correlated with changes in ADHD symptom severity.
4. Changes in QbCheck and CPT‐II will be correlated with changes in impairment and QoL.

## METHOD

### Participants

Inclusion criteria were individuals aged 6–25 years who are newly diagnosed with ADHD and seeking pharmacological treatments or with an existing diagnosis of ADHD seeking a change in their current ADHD medication regimen. All participants were clinically diagnosed with ADHD from a semi‐structured diagnostic interview with an experienced psychiatrist, using the Kiddie Schedule for Affective Disorders and Schizophrenia (Kaufman et al., 1997; Sonnby et al., 2015). During the clinical interview, developmental history relevant to ADHD, along with self‐reports and parent collateral on past and present ADHD symptoms across different settings and the severity of impairment, was collected. The exclusion criteria included co‐occurring clinical diagnoses (e.g., severe depression, severe anxiety, cerebral palsy, and other motor disorders), alcohol or substance abuse, limited proficiency in English, current use of prescription medications that could significantly affect test performance (e.g., neuroleptic and sedative medications), and concurrent audio‐visual sensory impairments.

### Materials

#### Neurocognitive outcomes

*QbCheck*. This online software uses laptops or computers with webcams and internet connections and offers two versions for different age groups (Qbtech, 2016). For children (ages 6–11), grey circles and grey circles with crosses are shown rapidly in a Go/No‐Go format. For adolescents/adults (ages 12–60), red and blue circles and squares are displayed in a matching pattern format (press the spacebar when the same shape and colour as the previous one appears). Stimuli are shown for 100 milliseconds for children and 200 milliseconds for adolescents/adults, with a total of 450 stimuli for children and 600 for adolescents/adults.

Responding to target stimuli by pressing the spacebar is required while ignoring non‐targets. QbCheck calculates five outcomes: MicroEvents (head movements via webcam), commission errors (CE—incorrect response to non‐targets), omission errors (OE—failure to respond to targets), reaction time (RT—mean time to respond) and reaction time variability (RTV—standard deviation of RT). The software compares individual performance to a normative group by age and gender, producing *Q*‐scores. Positive scores suggest below‐norm performance, possibly indicating ADHD, while negative scores indicate better‐than‐average performance (Qbtech, 2016; Ulberstad et al., 2020). CE reflects impulsivity, OE reflects inattention, and RT/RTV reflect sustained attention (Bezdjian et al., 2009; Fortenbaugh et al., 2017; Hasegawa et al., 2019; Perri et al., 2015, 2017; Robinson et al., 2020; Seli et al., 2012; Trommer et al., 1988).

*Conners' CPT‐II version 5.1 for windows*. This computerised test is designed for ages six and above and has been commonly used to measure inattention and impulsivity in ADHD research (Egeland & Kovalik‐Gran, 2010). Participants respond to a stimulus (random letters) on a black screen with white letters. They press the spacebar for each letter except for ‘X’. Outcomes assessed include CE, OE, RT and RTV. The test lasts 15–20 min.

#### Clinical outcomes

*ADHD symptom severity*. The SNAP Scale assessed the subjective opinions of parents or close individuals regarding the participants' ADHD symptoms (Swanson et al., 1999, 2001). The SNAP scale includes 18 behavioural descriptions (nine inattentive and nine hyperactive/impulsive) of ADHD core symptoms, rated on a four‐point Likert Scale from ‘Never (0)’ to ‘Very Often (3)’. ADHD symptom severity, as rated by the participants, was assessed using built‐in questionnaires within the QbCheck, matching the order and behavioural descriptions of the SNAP Scale.

*Functional impairment*. The treating psychiatrist used the ADHD Severity Clinical Global Impression (CGI; Alda & Serrano‐Troncoso, 2013; Busner & Targum, 2007) to quantify the functional impairment of ADHD symptoms. The CGI gauges recent ADHD symptom intensity in academic, home, social, and personal aspects. Impairment severity scores range from 1 (not at all ill) to 7 (very severely ill).

*Quality of life*. The participants' QoL was measured by the Paediatric Quality of Life Inventory (Varni et al., 2001). This questionnaire is widely used to gauge health‐related QoL in youths (Lee et al., 2016; Limbers et al., 2011). The questionnaire encompasses four domains: physical, emotional, social, and school. Respondents rate the extent of each statement's impact over the past month on a five‐point Likert scale, ranging from 0 (never a problem) to 4 (almost always a problem). Parents or close others also provided their opinions using the same scale. Responses were reversed on a 0 to 100 scale (e.g., 0 = 100, 4 = 0). Mean domain scores were computed, and an overall QoL score emerged from the average of all items. This system yields lower scores for reduced QoL.

#### Demographic and other measures

*Intellectual potential*. At baseline, participants' intellectual potential was evaluated using the Wechsler Abbreviated Scale of Intelligence‐Second Edition (WASI‐II; Wechsler, 2018). All four subtests (Vocabulary, Similarities, Block Design, and Matrix Reasoning) were utilised to calculate the estimated Full Scale Intellectual Quotient (FSIQ), assessing inherent cognitive ability. The tool is designed for individuals aged six to 90. Participants who had valid FSIQ results (within the last 2 years) were exempt from the assessment.

*Medication*. The participants' daily medication dosage and intake time were recorded on the day of the neuropsychological assessment. Following this, the medication information provided was cross‐referenced with the prescription, with consent from all participants and their treating clinician. All participants were instructed to adhere to their usual medication dosage on the assessment day, ensuring the assessment was scheduled within the medication's effective duration. To standardize the effects of various ADHD medications, a defined daily dosage rate (DDDR) was employed. We applied the DDDR based on the World Health Organization (WHO) Anatomical Therapeutic Chemical (ACT) classification system (WHO, 2023).

### Procedure

Participants were recruited from two different ADHD clinics in Melbourne, Australia. Inform consented participants were asked to attend two additional neuropsychological assessments apart from their standard care appointments. Eight participants voluntarily took an additional QbCheck assessment under the same conditions: three without medication and five on the same dosage. At baseline (i.e., before initiating their newly prescribed ADHD medication), each participant underwent objective assessments (intelligence estimation, QbCheck and Conners' CPT II), provided subjective ratings of the severity of their core ADHD symptoms (self and close other reported), and assessed their quality of life (self and close other reported). At their follow‐up appointment (i.e., after completing their titration process), participants were asked to repeat the CPT measures and subjectively report their ADHD symptom severity and QoL. The interval between baseline and follow‐up appointments ranged from 2 to 10 months. The order of data collection remained the same across all sessions as follows: (1) QbCheck ADHD symptoms self‐rating, (2) QbCheck test, (3) CPT‐II, (4) intelligence assessment (only at baseline or on a different day), and (5) questionnaires. A trainee neuropsychologist/researcher explained the QbCheck report to each participant and their close others (i.e., parents or partners) each session. All participants had their routine clinical assessments with their treating psychiatrist. The research obtained approval from The Royal Children's Hospital Melbourne Human Research Ethics Committee.

### Statistical power analysis

All power analyses used G*Power 3.1 (Faul et al., 2009).

#### Changes after treatment

The study was conducted during the COVID‐19 pandemic, and due to unforeseen barriers, the final sample size did not reach the intended *N* = 50 (see Supporting Information). A post‐hoc power analysis indicated that the achieved power of 0.89, given *N* = 34, Cohen's *d* = 0.3, and *α* = 0.05.

#### Test‐retest reliability (QbCheck)

An a priori power analysis indicated that a minimum of 27 participants was required to achieve sufficient power (1 − *β* = 0.8) for a *t*‐test, given Cohen's *d* = 0.3 and *α* = 0.05. Although the sample size was insufficient for full statistical power, the results provide preliminary evidence to evaluate the hypothesis.

#### Bivariate correlation

A prospective power analysis showed a minimum sample size of 23 participants to detect a significant correlation with sufficient statistical power. The analysis was based on the following parameters: a large effect size (*r* = 0.5), power (1 − *β* = 0.80), significance level (*α* = 0.05), and a one‐tailed correlation test.

### Methods of statistical analysis

Bayesian methods were used for all statistical analyses to minimize Type II errors and reduce the risk of inflated Type I error rates caused by the reliance on classical *p*‐value cutoffs in underpowered studies (van de Schoot et al., 2021). Using Bayesian methods, missing data was handled by estimation based on all available data, enabling the use of all collected data across time points. All data were analysed using JASP version 0.19.3 (JASP Team, 2025). Two statistical tests were used: (1) paired *t‐*tests to evaluate the likelihood of differences between baseline and post‐treatment, as well as the test‐retest validity of QbCheck measures, and (2) bivariate correlations to assess convergent validity between CPT‐II and QbCheck, as well as cross‐domain relationships between CPT measures and clinical outcomes.

## RESULTS

### Participants characteristics

Thirty‐four participants completed both baseline and follow‐up assessments. Table 1 summarises their demographic statistics. Two participants from the baseline did not attend the follow‐up assessment. One was no longer seeking treatment, and another could not participate in an in‐person session in the clinic due to work commitments.

**TABLE 1:** Participants' clinical and demographic characteristics means and standard deviations.

	Baseline			Follow‐up		
	Female	Male	Total	Female	Male	Total
Sex at birth	19	17	36	18	16	34
Age mean (SD)	16.18 (4.90)	16.53 (4.10)	16.35 (4.48)	16.47 (4.97)	16.83 (4.12)	16.64 (4.53)
FSIQ mean (SD)	109.89 (13.25)	106.82 (18.36)	107.92 (14.85)	108.83 (11.58)	106.56 (18.93)	107.77 (15.27)
DDDR mean^a^ (SD)	0.11 (0.32)	0.23 (0.48)	0.16 (0.40)	1.48 (0.65)	1.55 (0.67)	1.51 (0.65)
ADHD presentations						
Inattentive	5	7	12	5	6	11
Hyperactive/impulsive	‐	1	1	‐	1	1
Combined	14	9	23	13	9	22

Abbreviations: ADHD, Attention Deficit Hyperactivity Disorder; DDDR, defined daily dosage rate; FSIQ, Full‐Scale Intelligence Quotient; SD, standard deviation.

^a^ See Supporting Information S1: Table S1, for further details on ADHD medications.

### Psychometrics of outcome measures

Convergent Validity between QbCheck and CPT‐II. Table 2 presents the results of multiple Bayesian correlation analyses examining the relationships between QbCheck and Conner's CPT‐II variables. Overall, the results provide evidence of correlations between QbCheck and CPT‐II variables that are intended to measure the same constructs at each time point, including patterns of change following optimized ADHD medication. Only exception is the change in omission errors between QbCheck and CPT‐II, no correlation was found (BF_10_ = 0.21, *r* = 0.001, 95% CI [−0.33, 0.32]). See Table S2 for cross‐construct correlations, which show evidence for the null hypothesis between omission and commission errors in both QbCheck and CPT‐II. Also, provide evidence for discriminatory validity that no or weak correlation between two different constructs such MicroEventX and CPT‐II omission errors.

**TABLE 2:** Bayesian Pearson's *ρ* correlation coefficients between QbCheck and CPT‐II.

QbCheck	Conners CPT‐II	Pearson's *r*	BF_10_	95% CI	
Cross‐sectional at baseline (*n* = 36)					
Commission errors	Commission errors	0.36	1.95	0.03	0.60
Omission errors	Omission errors	0.43	5.98	0.11	0.65
Reaction time	Reaction time	0.53	46.58	0.23	0.72
Reaction time variability	Reaction time variability	0.66	2377.03	0.41	0.80
Cross‐sectional at follow‐up (*n* = 34)					
Commission errors	Commission errors	0.50	15.03	0.18	0.70
Omission errors	Omission errors	0.51	19.09	0.19	0.71
Reaction time	Reaction time	0.69	3088.98	0.43	0.82
Reaction time variability	Reaction time variability	0.68	2817.18	0.43	0.82
Changes between baseline and follow‐up					
Commission errors	Commission errors	0.33	1.19	−0.01	0.58
Omission errors	Omission errors	−0.001	0.21	−0.33	0.32
Reaction time	Reaction time	0.39	2.44	0.05	0.62
Reaction time variability	Reaction time variability	0.31	1.02	−0.03	0.57

*Note*: BF_10_ quantifies evidence for H_1_ (correlation) over H_0_ (no correlation). BF_10_ < 1 suggests support for H_0_. BF_10_ > 3–10 moderate evidence for correlation. BF_10_ > 10–30 strong evidence for correlation. BF_10_ > 100 extreme evidence for correlation.

Abbreviations: BF, Bayes factor; CI, credible interval; H_0_; null hypothesis; H_1_, alternative hypothesis.

Test Retest Reliability for QbCheck. Five Bayesian paired samples *t*‐tests were conducted in JASP (Version 0.19.3) using the default Cauchy prior (scale = 0.707) to compare QbCheck variables across two time points for participants on the same treatment regimen (either medication‐free or at the same dosage). Eight participants volunteered to retake the QbCheck test on a different day from baseline. Table 3 results provided evidence in favour of the null hypothesis for MicroEvent X, omission errors, and reaction time, suggesting no meaningful difference in scores between two time points. Bayesian paired samples *t*‐tests indicated anecdotal evidence for a difference in commission errors (BF_10_ = 1.72) and moderate evidence for a difference in response time variability (BF_10_ = 5.57).

**TABLE 3:** Bayesian paired sample *t*‐test for QbCheck measures.

Measure	Timepoint 1				Timepoint 2				SMD	BF_10_
	*M*	SD	95% CI		*M*	SD	95% CI			
MicroEventX	2.50	1.26	1.45	3.55	2.01	0.70	1.43	2.60	0.87	0.48
Commission error	1.66	1.09	0.75	2.57	0.64	0.61	0.13	1.15	2.13	1.72
Omission error	0.50	1.12	−0.44	1.44	0.54	0.86	−0.18	1.26	0.14	0.34
Reaction time	0.73	1.05	−0.15	1.60	0.81	1.15	−0.15	1.77	0.85	0.35
RTV	1.05	1.06	0.17	1.94	0.53	1.37	−0.62	1.67	1.68	5.57

*Note*: BF_10_ quantifies strengths of evidence for H_0_ (no difference) or H_1_ (difference exists). BF_10_ < 1 suggests support for H_0_. BF_10_ > 1–3 anecdotal evidence for H_1_. BF_10_ > 3–10 moderate evidence for H_1_. BF_10_ > 10–30 strong evidence for H_1_. BF_10_ > 100 extreme evidence for H_1_.

Abbreviations: BF, Bayes factor; CI, credible interval; H_0_, null hypothesis; H_1_, alternative hypothesis; RTV, reaction time variability.

Inter‐rater Reliability for Clinical Measures. Table 4 presents the results of multiple Bayesian correlation analyses examining the relationships between self‐ and informant‐reported clinical measures (i.e., ADHD symptoms and QoL) at each time point, including patterns of change over time. Overall, the results provide evidence of no correlation between self‐ and informant‐reported ADHD symptom ratings and QoL at baseline or in their patterns of change. At follow‐up, there was moderate evidence for a strong positive correlation (BF_10_ = 3.25, *r* = 0.51, 95% CI [0.07, 0.75]) between self‐ and informant‐rated QoL and weak evidence for a moderate positive correlation (BF_10_ = 2.04, *r* = 0.46, 95% CI [0.02, 0.72]) between self‐ and informant‐rated ADHD hyperactive/impulsive symptom ratings. This suggests cross‐sectional consistency between self‐ and informant‐reported clinical measures at follow‐up.

**TABLE 4:** Bayesian Pearson's *ρ* correlation coefficients between self and informant reports.

Self and informant^a^	*n*	Pearson's *r*	BF_10_	95% CI	
Cross‐sectional at baseline					
ADHD symptom total	36	0.23	0.44	−0.18	0.55
ADHD inatttative symptom	36	0.27	−0.44	0.32	0.27
ADHD hyperactivity/impulsivity symptom	36	0.26	0.49	−0.19	0.59
QoL total	22	0.28	0.55	−0.16	0.60
Cross‐sectional at follow‐up					
ADHD symptom total	34	0.30	0.60	−0.15	0.62
ADHD inatttative symptom	34	0.24	0.46	−0.20	0.58
ADHD hyperactivity/impulsivity symptom	34	0.46	2.04	0.02	0.72
QoL total	20	0.51	3.25	0.07	0.75
Changes between baseline and follow‐up					
ADHD symptom total	34	0.21	0.39	−0.23	0.55
ADHD inatttative symptom	34	0.22	0.41	−0.23	0.56
ADHD hyperactivity/impulsivity symptom	34	0.27	0.52	−0.18	0.60
QoL total	19	0.24	0.45	−0.22	0.59

*Note*: BF_10_ quantifies evidence for H_1_ (correlation) over H_0_ (no correlation). BF_10_ < 1 suggests support for H_0_. BF_10_ > 3–10 moderate evidence for correlation. BF_10_ > 10–30 strong evidence for correlation. BF_10_ > 100 extreme evidence for correlation.

Abbreviations: ADHD, Attention Deficit Hyperactive Disorder; BF, Bayes factor; CI, credible interval; H_0_, null hypothesis; H_1_, alternative hypothesis; QoL, quality of life.

^a^Informants consisted of either a parent or a partner.

### Changes between baseline and follow‐up

Table 5 summarizes the results of Bayesian paired samples *t*‐tests (default Cauchy prior, scale = 0.707) assessing the likelihood of differences between baseline and follow‐up. The analysis provided moderate to extreme evidence in favour of the alternative hypothesis for both self‐ and informant‐rated ADHD symptoms and clinician‐rated impairment severity, indicating a high probability of reduction of severity in these clinical measures. A Bayesian paired samples *t*‐test comparing baseline and follow‐up informant‐rated QoL (BF_10_ = 1.81) provided weak evidence for a difference. However, the 95% credible interval [0.952, −0.002] suggests the effect may be small or uncertain. Similarly, a Bayesian paired samples *t*‐test for self‐rated QoL (BF_10_ = 0.67, 95% CI [−0.60, −0.06]) provided evidence supporting no difference between the two time points.

**TABLE 5:** Means and standard deviations and changes between baseline and follow‐up.

Measure	Baseline				Follow‐up				SMD^b^	95% CI		BF_10_
	*M*	SD	95% CI		*M*	SD	95% CI			LL	UL	
QbCheck												
*n*	36				34							
MicroEventX	2.06	1.37	1.60	2.52	1.25	1.34	0.76	1.74	27.00	0.17	0.89	14.88
CE	1.25	1.4	0.81	1.70	0.31	1.25	−0.13	0.74	6.27	0.28	1.02	91.89
OE	0.83	1.11	0.45	1.20	0.2	1.3	−0.25	0.66	3.32	0.15	0.86	10.57
RT	1.01	1.03	0.66	1.36	0.74	1	0.40	1.09	9.00	0.01	0.68	1.38
RTV	1.26	1.25	0.84	1.69	0.19	1.23	−0.24	0.62	53.50	0.64	1.51	84,074.67
Conners CPT‐II												
*n*	36				34							
CE	18.56	6.84	16.24	20.87	13.79	8.33	10.89	16.70	3.20	0.32	1.08	201.49
OE	15.39	29.71	5.34	25.44	8.56	19.81	1.65	15.47	0.69	−0.06	0.60	0.66
RT	447.96	131.14	393.93	482.00	422.95	102.15	387.30	458.59	0.86	−0.16	0.49	0.30
RTV	23.77	28.62	14.09	33.45	14.34	13.88	9.50	19.18	0.64	0.02	0.70	1.64
Impairment												
*n*	36				34							
CGI	5.56	0.61	5.35	5.76	2.59	0.74	2.33	2.85	21.82	^c^	^c^	7.265 × 10 + 16
Self‐rated ADHD symptom												
*n*	36				34							
Inattention	1.89	0.68	1.66	2.12	1.09	0.52	0.91	1.27	5.04	0.64	1.51	79,239.68
Hyperactivity/impulsivity	1.36	0.76	1.10	1.61	0.99	0.65	0.76	1.21	3.39	−1.05	−0.07	3.31
Total	1.62	0.63	1.41	1.83	1.04	0.53	0.85	1.22	5.87	0.52	1.34	7305.75
Informant‐rated ADHD symptom^a^												
*n*	24				21							
Inattention	2.32	0.48	2.12	2.52	1.40	0.83	1.02	1.78	2.61	0.81	2.10	22,504.92
Hyperactivity/impulsivity	1.15	0.85	0.79	1.51	0.63	0.61	0.36	0.91	2.11	0.44	1.51	254.52
Total	1.73	0.55	1.50	1.97	1.02	0.61	0.74	1.30	11.90	0.77	2.04	14,979.55
Self‐rated quality of life												
*n*	36				34							
Physical	74.22	18.68	67.90	80.54	77.43	17.90	71.18	83.67	4.14	−0.41	0.23	0.21
Emotional	53.75	18.56	47.47	60.03	57.40	16.12	51.78	63.03	1.50	−0.44	0.20	0.24
Social	78.15	21.41	70.91	85.39	80.78	15.71	75.30	86.27	0.46	−0.36	0.28	0.19
School	39.17	23.69	31.15	47.18	50.49	18.18	44.15	56.83	2.06	−0.73	−0.05	2.36
Self‐total	61.32	15.75	55.99	66.65	66.53	12.58	62.14	70.91	1.64	−0.60	−0.06	0.67
Informant‐rated quality of life^a^												
*n*	22				20							
Physical	72.95	24.55	62.07	83.84	79.06	21.00	69.24	88.89	1.72	−0.60	0.06	0.67
Emotional	55.11	22.27	45.24	64.99	65.81	21.26	55.86	75.76	10.56	−0.83	0.09	0.87
Social	82.12	17.15	74.52	89.72	80.83	19.11	71.89	89.78	0.65	−1.50	−0.36	71.13
School	46.52	12.17	41.12	51.91	58.50	25.64	46.50	70.50	0.89	−0.48	0.38	0.25
Total	64.18	13.90	58.01	70.34	71.05	17.22	62.99	79.11	2.07	0.952	−0.002	1.81

*Note*: BF_10_ quantifies strengths of evidence for H_0_ (no difference) or H_1_ (difference exists). BF_10_ < 1 suggests support for H_0_. BF_10_ > 1–3 anecdotal evidence for H_1_. BF_10_ > 3–10 moderate evidence for H_1_. BF_10_ > 10–30 strong evidence for H_1_. BF_10_ > 100 extreme evidence for H_1_.

Abbreviations: ADHD, Attention Deficit Hyperactive Disorder; BF, Bayes factor; CE, commission error; CGI, Clinical Global Impression; CI, credible interval; CPT, continuous performance test; H_0_, null hypothesis; H_1_, alternative hypothesis; OE, omission errors; RT, reaction time; RTV, reaction time variability.

^a^ Informants consisted of either a parent or a partner.

^b^ Standard Mean Difference (SMD) was manually calculated based on Mean difference between baseline and follow‐up/standard deviation of the differences between paired observations.

^c^ The 95% CI could not be estimated for CGI due to the posterior distribution being highly concentrated near zero.

Results for QbCheck variables showed moderate to extreme evidence supporting changes in all measures (MicroEventX, CE, OE, and RTV), except for RT, which showed weak evidence for a difference (BF_10_ = 1.38, 95% CI [0.01, 0.68]). Conners CPT‐II variables provided extremely strong evidence for a change in CE, weak evidence for RTV, and insufficient evidence to support changes in OE and RT.

### Cross‐domain relationships of changes between CPT and clinical measures

Multiple Bayesian correlation analyses were conducted to examine the hypothesised longitudinal cross‐domain relationships. Additionally, correlations were only investigated among variables that changed over time, as identified in the previous Bayesian paired‐samples *t*‐tests. Results presented in Table 6 identified two correlations supported by weak evidence, suggesting positive moderate associations between QbCheck MicroEventX and informant‐rated ADHD total symptom severity (BF_10_ = 2.40, *r* = 0.47, 95% CI [0.04, 0.73]) and between CPT‐II commission errors and self‐rated ADHD total symptom severity (BF_10_ = 2.44, *r* = 0.39, 95% CI [0.05, 0.62]). No evidence was found to support any other cross‐domain relationships.

**TABLE 6:** Bayesian Pearson's *ρ* correlation coefficients between changes in CPT and clinical measures.

CPT measures	Clinical measures	Pearson's *r*	BF_10_	95% CI	
Changes QbCheck and self‐rated ADHD symptoms (*n* = 34)					
MicroEventX	Hyperactive/impulsivity	0.09	0.24	−0.25	0.40
Commission errors	Hyperactive/impulsivity	0.13	0.28	−0.21	0.43
Omission errors	Inattention	0.28	0.54	−0.17	0.60
Reaction time	Inattention	0.25	0.55	−0.10	0.52
Reaction time variability	Inattention	0.26	0.59	−0.09	0.53
MicroEventX	Total	0.09	0.24	−0.25	0.40
Commission errors	Total	0.14	0.29	−0.20	0.44
Omission errors	Total	0.25	0.57	−0.09	0.53
Reaction time	Total	0.27	0.69	−0.07	0.54
Reaction time variability	Total	0.26	0.50	−0.18	0.59
Changes QbCheck and Informant‐R ated ADHD symptoms (*n* = 21)					
MicroEventX	Hyperactive/impulsivity	0.41	1.33	−0.03	0.69
Commission errors	Hyperactive/impulsivity	0.20	0.38	−0.24	0.55
Omission errors	Inattention	0.28	0.54	−0.17	0.60
Reaction time	Inattention	0.24	0.45	−0.20	0.58
Reaction time variability	Inattention	0.37	0.99	−0.07	0.66
MicroEventX	Total	0.47	2.40	0.04	0.73
Commission errors	Total	0.28	0.53	−0.17	0.60
Omission errors	Total	0.13	0.32	−0.30	0.50
Reaction time	Total	0.13	0.31	−0.30	0.50
Reaction time variability	Total	−0.09	0.30	−0.49	0.35
Changes QbCheck and clinician‐rated impairment (*n* = 34)					
MicroEventX	CGI	0.23	0.50	−0.11	0.51
Commission errors	CGI	0.05	0.22	−0.28	0.37
Omission errors	CGI	−0.09	0.24	−0.40	0.25
Reaction time	CGI	−0.09	0.24	−0.40	0.25
Reaction time variability	CGI	0.01	0.21	−0.32	0.33
Changes QbCheck and informant‐rated QoL (*n* = 19)					
MicroEventX	Total	−0.09	0.30	−0.49	0.35
Commission errors	Total	0.18	0.37	−0.28	0.55
Omission errors	Total	−0.15	0.34	−0.53	0.30
Reaction time	Total	−0.12	0.32	−0.51	0.33
Reaction time variability	Total	−0.09	0.30	−0.49	0.35
MicroEventX	School function	−0.11	0.31	−0.50	0.32
Commission errors	School function	0.37	0.94	−0.08	0.67
Omission errors	School function	0.17	0.35	−0.28	0.54
Reaction time	School function	0.04	0.28	−0.38	0.44
Reaction time variability	School function	−0.17	0.35	−0.54	0.27
Conners CPT‐II and self‐rated ADHD symptoms (*n* = 34)					
Commission errors	Hyperactive/impulsivity	0.33	1.19	−0.01	0.58
Reaction time variability	Inattention	−0.001	0.21	−0.33	0.32
Commission errors	Total	0.39	2.44	0.05	0.62
Reaction time variability	Total	0.31	1.02	−0.03	0.57
Conners CPT‐II and informant‐rated ADHD symptoms (*n* = 21)					
Commission errors	Hyperactive/impulsivity	0.31	0.65	−0.14	0.62
Reaction time variability	Inattention	0.17	0.35	−0.26	0.53
Commission errors	Total	0.16	0.34	−0.27	0.53
Reaction time variability	Total	0.28	0.56	−0.16	0.61
Conners CPT‐II and informant‐rated QoL (*n* = 19)					
Commission errors	Total	−0.30	0.58	−0.63	0.17
Reaction time variability	Total	0.16	0.35	−0.29	0.54
Commission errors	School function	−0.14	0.33	−0.52	0.30
Reaction time variability	School function	−0.10	0.30	−0.49	0.33
Conners CPT‐II and clinician‐rated impairment (*n* = 34)					
Commission errors	CGI	0.30	0.86	−0.05	0.56
Reaction time variability	CGI	0.28	0.73	−0.06	0.55

*Note*: BF_10_ quantifies evidence for H_1_ (correlation) over H_0_ (no correlation). BF_10_ < 1 suggests support for H_0_. BF_10_ > 1–3 anecdotal evidence for H_1_. BF_10_ > 3–10 moderate evidence for correlation. BF_10_ > 10–30 strong evidence for correlation. BF_10_ > 100 extreme evidence for correlation.

Abbreviations: ADHD, Attention Deficit Hyperactive Disorder; BF, Bayes factor; CE, commission error; CGI, Clinical Global Impression; CI, credible interval; CPT, continuous performance test; H_0_, null hypothesis; H_1_, alternative hypothesis; OE, omission errors; QoL, quality of life; RT, reaction time; RTV, reaction time variability.

## DISCUSSION

This study aimed to evaluate the psychometric properties of QbCheck in measuring changes during ADHD pharmacological treatment and to explore the relationship between longitudinal changes in QbCheck results and clinical outcomes across three domains: ADHD core symptoms, impairment, and QoL. In the assessment of QbCheck's psychometric properties and its ability to quantify change during treatment, Bayesian correlation analyses (Table 2) revealed expected cross‐sectional and longitudinal correlations between QbCheck and Conners' CPT‐II variables measuring the same constructs, supporting QbCheck's convergent validity in assessing neurocognitive functions related to impulsivity and attention. A notable exception was the absence of correlation for changes in omission errors between QbCheck and CPT‐II. Test‐retest reliability of QbCheck variables (Table 3) demonstrated stability at two different time points for most variables, with no significant differences observed in MicroEventX, omission errors, or reaction time. However, commission errors and reaction time variability appeared less reliable than other variables. QbCheck showed moderate to extreme evidence for changes in most variables (Table 5), highlighting its ability to detect changes during ADHD treatment. However, reaction time showed only weak evidence for a difference, suggesting that response speed may be less responsive to treatment than other measures. In contrast, the CPT‐II results provided strong evidence for reductions in commission errors, weak evidence for reaction time variability, and no evidence for changes in omission errors or reaction time, indicating that CPT‐II measures may be less sensitive in capturing changes compared to QbCheck. Overall, both QbCheck and CPT‐II show potential in quantifying changes during ADHD treatment.

Additionally, Table 4 presents the inter‐rater reliability between self‐ and informant‐reported ADHD symptoms and QoL. At baseline, no significant correlations were observed, indicating a lack of alignment between raters' reports. However, at follow‐up, a strong positive correlation between QoL and hyperactive/impulsive ADHD symptoms suggested better agreement between raters in reporting these clinical outcomes. Notably, there was no agreement in changes between self‐ and informant‐reported variables, indicating inconsistent reporting of change across raters.

To explore the longitudinal cross‐domain relationship between CPT and clinical variables, Bayesian Paired Samples *t*‐tests (Table 5) indicated that clinical measures showed changes between baseline and follow‐up. There was moderate to extreme evidence for reductions in self‐ and informant‐rated ADHD symptoms, as well as clinician‐rated impairment. Informant‐rated QoL showed weak evidence for change over time, primarily driven by the school function subcategory. However, self‐rated QoL showed no evidence to support differences. Bayesian longitudinal cross‐domain correlations (Table 6) showed weak evidence for moderate associations between QbCheck MicroEventX and informant‐rated ADHD total symptoms, as well as between CPT‐II commission errors and self‐rated ADHD total symptoms. Unlike hypothesis, 2.2, no other longitudinal cross‐domain relationships were supported, indicating limited overlap between the CPT and clinical variables. This suggests that QbCheck and CPT‐II capture distinct aspects of changes during ADHD pharmacological treatment rather than a proxy measure for clinical outcomes.

Changes in CPT variables with ADHD medication have been consistently reported in the literature (Emser et al., 2018; Lee et al., 2022), along with the advantage of CPT in providing reliable measures that are less prone to bias compared to subjective reports (Cedergren et al., 2022; Slobodin & Davidovitch, 2022). Weak or inconsistent inter‐rater agreement in assessing ADHD symptom severity, impairment, and quality of life is also consistent with previous findings (Arrondo et al., 2024; Bussing et al., 2008; Dallos et al., 2017; Lee et al., 2022; Martin et al., 2007; Moraes et al., 2020; Narad et al., 2015; Schneider et al., 2020; Söderström et al., 2014; Sumner et al., 2010). The novel contribution of this study is its simultaneous validation of two CPT variables and assessment of clinical measure reliability. A key strength is its comprehensive approach to addressing the often‐overlooked inconsistencies across ADHD assessment methods.

Limited or no correlations between CPT and clinical variables align with previous findings (Cedergren et al., 2022; Summer et al., 2010; Vaughn et al., 2011). Unlike earlier QbTest validation studies focused on cross‐sectional validity (Cedergren et al., 2022; Ulberstad et al., 2020), this study examines relationships of patterns of changes in two time points, providing additional insights into ADHD treatment and assessment. Two variables showed similar patterns of change: QbCheck MicroEventX with informant‐rated ADHD total symptoms and CPT‐II commission errors with self‐rated ADHD total symptoms. Given that MicroEventX objectively measures head movement, its correlation with informant‐rated ADHD symptoms is expected, as increased hyperactivity may be observable by others. However, the positive correlation between CPT‐II commission errors and self‐reported ADHD total symptoms suggests that individuals who perceive higher symptom severity made more impulsive responses. On the other hand, changes in QbCheck's neurocognitive variables OE, CE, RT, and RTV cannot be a proxy measure for changes in any other clinical variables. The results suggest that CPT outcomes cannot be a proxy measure for behaviourally observed clinical measures. However, they also highlight the importance of assessing changes in both cognitive and behavioural aspects (Snyder et al., 2015; Yap et al., 2021). This unique finding underscores the need for future ADHD treatment research to incorporate comprehensive measurement approaches in their outcome evaluations.

The lack of correlation between QbCheck and clinical variables may reflect the neurocognitive heterogeneity of ADHD. Rather than a single or a set of neurocognitive challenges, individuals with ADHD exhibit diverse strengths and differences across multiple domains (Coghill, Hayward, et al., 2014; Coghill, Seth, & Matthews, 2014; Fair et al., 2012; Nigg et al., 2005; Roberts et al., 2017). Both CPT‐II and QbCheck assess only specific neurocognitive functions, primarily sustained attention and response inhibition, but do not capture broader processes like working memory and cognitive flexibility (Kofler et al., 2024). For example, studies indicate a potential link betweeen working memory changes and shifts in ADHD symptoms (Arjona et al., 2023; Karalunas & Huang‐Pollock, 2011; Kollins et al., 2020), underscoring the need for a broader assessment of neurocognitive functions in ADHD research. Pharmacological effects on neurocognition and behaviour may also vary (Bidwell et al., 2011). Randomized Control Trials of methylphenidate at different doses found that neurocognitive performance peaked at medium doses, while symptom reduction was greatest at higher doses (Barkley, 1997; Coghill et al., 2007; Solanto et al., 2009). This suggests that neurocognitive measures, including CPT, do not always align directly with behavioural symptom changes (Bijlenga et al., 2015). Thus, while CPT performance may not correlate with clinical variables, this does not mean neurocognition is unrelated to ADHD characteristics. Instead, it highlights the need for broader neurocognitive assessments in ADHD research to improve understanding of symptom trajectories and treatment outcomes (Wigal et al., 2011).

While this study offers valuable insights, several limitations should be considered. The study design did not control for extraneous variables like sleep (Gruber et al., 2007; Morash‐Conway et al., 2017; Owens et al., 2013), anxiety (Bloemsma et al., 2013; Prevatt et al., 2015), mood (Sandstrom et al., 2021; Skirrow et al., 2009), and fatigue. The limited scope of neurocognitive measures and the absence of a control group, along with variations in age, medication type, and dosage, restrict the generalizability. However, the variability in the sample aided in testing the clinical utility of QbCheck and identifying patterns of relationships (Brocki et al., 2010).

In conclusion, QbCheck is a valid tool for assessing certain aspects of neurocognitive changes following ADHD pharmacological treatment but should not replace clinical outcome measures. The findings highlight that relying on a single outcome measure may not fully capture treatment effects. Notably, changes in QbCheck movement scores (MicroEventX) align with informant‐rated ADHD symptom severity, suggesting its value as an alternative when multi‐source clinical assessments are impractical. Additionally, the low inter‐rater reliability in clinical outcomes suggests that QbCheck could be an objective indicator of treatment effects when discrepancies arise between reporters.

Despite the clinical utility of CPT results, the long‐term clinical significance of these neurocognitive changes remains unclear. Current findings provide limited insight into how improvements in neurocognitive function translate into meaningful behavioural or functional changes over a two‐ to 10‐month follow‐up period. To address this gap, future research should explore the extended impact of neurocognitive changes on functional outcomes, such as academic performance, occupational success, and social interactions. A deeper understanding of these relationships will improve assessments of ADHD pharmacological treatment's biopsychosocial impact and refine treatment approaches for more meaningful outcomes.

## AUTHOR CONTRIBUTIONS

**Seungjae Lee**: Conceptualization; data curation; formal analysis; investigation; methodology; project administration; resources; validation; visualization; writing—original draft; writing—review and editing. **Renee Testa**: Conceptualization; methodology; resources; supervision. **Beth Johnson**: Methodology; resources; supervision; writing—review and editing. **David Coghill**: Conceptualization; data curation; formal analysis; methodology; project administration; supervision; writing—review and editing.

## CONFLICT OF INTEREST STATEMENT

Seungjae Lee, Renee Testa and Beth Johnson have no disclosures. David Coghill received royalties from the Oxford University Press, honoraria from Takeda, Medice, and Novartis, and research funding from the Australasia ADHD Professionals Association and the National Health and Medical Research Council, unrelated to the work described here. This research received no specific grant from funding agencies in the public, commercial, or not‐for‐profit sectors.

## ETHICAL CONSIDERATIONS

The research obtained approval from The Royal Children's Hospital Melbourne Human Research Ethics Committee.

## Supporting information

Supporting Information S1

## Data Availability

The data that support the findings of this study are available on request from the corresponding author. The data are not publicly available due to privacy or ethical restrictions.
