# Supplementary material for: A comprehensive longitudinal analysis of changes during Attention Deficit Hyperactive Disorder pharmacological treatments: Relationships between clinical measures, QbCheck and Conners CPT‐II
Source: JCPP Adv. 2025 Jun 20;6(1):e70021. doi: 10.1002/jcv2.70021 (PMC12973167; doi:10.1002/jcv2.70021)
Supplement: Supplementary file 1 — Supporting Information S1 [file JCV2-6-e70021-s001.docx]

**A Comprehensive Longitudinal Analysis of Changes during ADHD Pharmacological Treatments: Relationships between Clinical Measures, QbCheck, and Conners CPT-II.**

## Supporting Information

**Power and Data Analysis**

**Outcome Changes After Treatment.** A prospective power analysis was conducted using G*Power 3.1 (Faul et al., 2009) to determine the minimum sample size required for a mixed-design two-way repeated measures ANOVA. The analysis included two factors: time (within-subjects) and sex assigned at birth (between-subjects). A medium-to-large effect size (Cohen’s *d* = .30) was chosen based on Cohen’s guidelines (as cited in Correll, 2019). The significance level was set at α = 0.05, and power (*1*−*β*) at .80. The study included two groups (male and female), assuming sphericity (*ϵ* = 1) and a moderate correlation (*r* = 0.5) among repeated measures, as commonly used in RCTs for ADHD medication studies (Dittner et al., 2014). Power analysis indicated that 24 participants per group (N = 48 total) were required to detect main effects and interactions with sufficient power. However, the final sample size (*N* = 34) resulted in lower power (achieved power = 0.67) for the mixed-design ANOVA.

**Table S1.**

*Frequency and Description of Participants' Medications*

|  |  |  | Baseline | | | Follow-up | | |
| --- | --- | --- | --- | --- | --- | --- | --- | --- |
|  |  |  | F | M | Total | F | M | Total |
| ADHD Medication Status DDDR | | |  |  |  |  |  |  |
| Free | | | 17 | 12 | 29 | 1 | 1 | 2 |
| Lisdexamfetamine 0.50 | | | - | 1 | 1 | - | - | - |
| Lisdexamfetamine 0.67 | | | - | 1 | 1 | - | - | - |
| Lisdexamfetamine 1.00 | | | 2 | 1 | 3 | 4 | 3 | 7 |
| Lisdexamfetamine 1.33 | | | - | - | - | 1 | 1 | 2 |
| Lisdexamfetamine 1.67 | | | - | 1 | 1 | 2 | 2 | 4 |
| Lisdexamfetamine 2.00 | | | - | - | - | 3 | 3 | 6 |
| Lisdexamfetamine 2.33 | | | - | - | - | 3 | 1 | 4 |
| Dexamphetamine 0.33 | | | - | - | - | 3 | 1 | 4 |
| Dexamphetamine 0.67 | | | - | - | - | - | 2 | 2 |
| Methylphenidate 0.33 | | | - | - | - | 1 | - | 1 |
| Methylphenidate 0.67 | | | - | - | - | - | 1 | 1 |
| MethylphenidateLA 1.00 | | | - | - | - | 1 | - | 1 |
| Concerta 1.80 | | | - | - | - | - | 1 | 1 |
| Concerta 2.60 | | | - | - | - | - | 1 | 1 |
| Guanfacine 0.33 | | | - | 1 | 1 | - | 2 | 2 |
| Other medications | | |  |  |  |  |  |  |
| Bupropion | | | - | 1 | 1 | - | - | - |
| Fluoxetine | | | 1 | - | 1 | - | - | - |
| Clonidine | | | - | - | - | 1 | - | 1 |
| Thyroxine | | | - | - | - | - | 1 | 1 |

| **Table S2.**  *Bayesian Pearson’s ρ Correlation Coefficients Between QbCheck and CPT-II for all Variables* | | | | | | |
| --- | --- | --- | --- | --- | --- | --- |
| **Bayesian Pearson Correlations** | |  |  |  |  |  |
|  |  | **n** | **Pearson's r** | **BF₁₀** | **Lower 95% CI** | **Upper 95% CI** |
| **T1_QB_MicroEventX** | **T1_QB_CE** | **36** | **0.334** | **1.388** | **0.003** | **0.580** |
| **T1_QB_MicroEventX** | **T1_QB_OE** | **36** | **0.229** | **0.493** | **-0.105** | **0.502** |
| **T1_QB_MicroEventX** | **T1_QB_RT** | **36** | **-0.088** | **0.235** | **-0.390** | **0.238** |
| **T1_QB_MicroEventX** | **T1_QB_RTV** | **36** | **0.263** | **0.657** | **-0.071** | **0.528** |
| **T1_QB_MicroEventX** | **T2_QB_MicroEventX** | **34** | **0.582** | **109.711** | **0.284** | **0.755** |
| **T1_QB_MicroEventX** | **T2_QB_CE** | **34** | **0.272** | **0.680** | **-0.072** | **0.541** |
| **T1_QB_MicroEventX** | **T2_QB_OE** | **34** | **0.403** | **3.101** | **0.068** | **0.635** |
| **T1_QB_MicroEventX** | **T2_QB_RT** | **34** | **-0.131** | **0.278** | **-0.433** | **0.208** |
| **T1_QB_MicroEventX** | **T2_QB_RTV** | **34** | **0.209** | **0.420** | **-0.134** | **0.494** |
| **T1_QB_MicroEventX** | **C_QB_MicroEventX** | **34** | **0.416** | **3.762** | **0.082** | **0.644** |
| **T1_QB_MicroEventX** | **C_QB_CE** | **34** | **-0.008** | **0.214** | **-0.331** | **0.318** |
| **T1_QB_MicroEventX** | **C_QB_OE** | **34** | **-0.353** | **1.588** | **-0.600** | **-0.012** |
| **T1_QB_MicroEventX** | **C_QB_RT** | **34** | **0.003** | **0.213** | **-0.322** | **0.327** |
| **T1_QB_MicroEventX** | **C_QB_RTV** | **34** | **0.031** | **0.216** | **-0.298** | **0.351** |
| **T1_QB_MicroEventX** | **T1_CPT_II_OE_Value** | **36** | **0.269** | **0.698** | **-0.064** | **0.532** |
| **T1_QB_MicroEventX** | **T1_CPT_II_CE_Value** | **36** | **0.330** | **1.331** | **-0.001** | **0.577** |
| **T1_QB_MicroEventX** | **T1_CPT_II_Hit RT_Value** | **36** | **-0.032** | **0.211** | **-0.343** | **0.288** |
| **T1_QB_MicroEventX** | **T1_CPT_II_RTV_Value** | **36** | **0.222** | **0.469** | **-0.112** | **0.497** |
| **T1_QB_MicroEventX** | **T2_CPT_II_OE_Value** | **34** | **0.366** | **1.875** | **0.027** | **0.609** |
| **T1_QB_MicroEventX** | **T2_CPT_II_CE_Value** | **34** | **0.370** | **1.970** | **0.031** | **0.612** |
| **T1_QB_MicroEventX** | **T2_CPT_II_Hit RT_Value** | **34** | **0.115** | **0.261** | **-0.223** | **0.420** |
| **T1_QB_MicroEventX** | **T2_CPT_II_RTV_Value** | **34** | **0.123** | **0.269** | **-0.215** | **0.426** |
| **T1_QB_MicroEventX** | **C_CPT_II_OE_Value** | **34** | **0.095** | **0.245** | **-0.241** | **0.404** |
| **T1_QB_MicroEventX** | **C_CPT_II_CE_Value** | **34** | **-0.134** | **0.280** | **-0.435** | **0.206** |
| **T1_QB_MicroEventX** | **C_CPT_II_Hit RT_Value** | **34** | **-0.119** | **0.265** | **-0.423** | **0.219** |
| **T1_QB_MicroEventX** | **C_CPT_II_RTV_Value** | **34** | **0.231** | **0.488** | **-0.113** | **0.510** |
| **T1_QB_CE** | **T1_QB_OE** | **36** | **0.581** | **159.179** | **0.295** | **0.751** |
| **T1_QB_CE** | **T1_QB_RT** | **36** | **0.177** | **0.346** | **-0.155** | **0.461** |
| **T1_QB_CE** | **T1_QB_RTV** | **36** | **0.597** | **253.061** | **0.316** | **0.762** |
| **T1_QB_CE** | **T2_QB_MicroEventX** | **34** | **0.321** | **1.103** | **-0.021** | **0.577** |
| **T1_QB_CE** | **T2_QB_CE** | **34** | **0.523** | **26.401** | **0.209** | **0.717** |
| **T1_QB_CE** | **T2_QB_OE** | **34** | **0.611** | **248.705** | **0.323** | **0.774** |
| **T1_QB_CE** | **T2_QB_RT** | **34** | **0.227** | **0.475** | **-0.117** | **0.507** |
| **T1_QB_CE** | **T2_QB_RTV** | **34** | **0.598** | **170.119** | **0.306** | **0.766** |
| **T1_QB_CE** | **C_QB_MicroEventX** | **34** | **-0.078** | **0.234** | **-0.390** | **0.256** |
| **T1_QB_CE** | **C_QB_CE** | **34** | **0.493** | **14.227** | **0.172** | **0.697** |
| **T1_QB_CE** | **C_QB_OE** | **34** | **-0.197** | **0.389** | **-0.484** | **0.146** |
| **T1_QB_CE** | **C_QB_RT** | **34** | **-0.039** | **0.218** | **-0.357** | **0.291** |
| **T1_QB_CE** | **C_QB_RTV** | **34** | **0.035** | **0.217** | **-0.294** | **0.355** |
| **T1_QB_CE** | **T1_CPT_II_OE_Value** | **36** | **0.461** | **9.755** | **0.145** | **0.670** |
| **T1_QB_CE** | **T1_CPT_II_CE_Value** | **36** | **0.360** | **1.949** | **0.031** | **0.599** |
| **T1_QB_CE** | **T1_CPT_II_Hit RT_Value** | **36** | **0.357** | **1.878** | **0.028** | **0.597** |
| **T1_QB_CE** | **T1_CPT_II_RTV_Value** | **36** | **0.500** | **21.113** | **0.191** | **0.697** |
| **T1_QB_CE** | **T2_CPT_II_OE_Value** | **34** | **0.290** | **0.804** | **-0.054** | **0.554** |
| **T1_QB_CE** | **T2_CPT_II_CE_Value** | **34** | **0.184** | **0.359** | **-0.158** | **0.474** |
| **T1_QB_CE** | **T2_CPT_II_Hit RT_Value** | **34** | **0.417** | **3.812** | **0.083** | **0.645** |
| **T1_QB_CE** | **T2_CPT_II_RTV_Value** | **34** | **0.370** | **1.984** | **0.032** | **0.612** |
| **T1_QB_CE** | **C_CPT_II_OE_Value** | **34** | **0.405** | **3.190** | **0.070** | **0.636** |
| **T1_QB_CE** | **C_CPT_II_CE_Value** | **34** | **0.117** | **0.263** | **-0.221** | **0.422** |
| **T1_QB_CE** | **C_CPT_II_Hit RT_Value** | **34** | **0.146** | **0.296** | **-0.194** | **0.445** |
| **T1_QB_CE** | **C_CPT_II_RTV_Value** | **34** | **0.442** | **5.718** | **0.112** | **0.662** |
| **T1_QB_OE** | **T1_QB_RT** | **36** | **0.568** | **110.218** | **0.277** | **0.743** |
| **T1_QB_OE** | **T1_QB_RTV** | **36** | **0.713** | **19053.142** | **0.479** | **0.835** |
| **T1_QB_OE** | **T2_QB_MicroEventX** | **34** | **0.078** | **0.234** | **-0.256** | **0.390** |
| **T1_QB_OE** | **T2_QB_CE** | **34** | **0.240** | **0.524** | **-0.104** | **0.517** |
| **T1_QB_OE** | **T2_QB_OE** | **34** | **0.656** | **1039.926** | **0.385** | **0.802** |
| **T1_QB_OE** | **T2_QB_RT** | **34** | **0.288** | **0.789** | **-0.056** | **0.553** |
| **T1_QB_OE** | **T2_QB_RTV** | **34** | **0.493** | **14.264** | **0.172** | **0.697** |
| **T1_QB_OE** | **C_QB_MicroEventX** | **34** | **0.074** | **0.232** | **-0.260** | **0.387** |
| **T1_QB_OE** | **C_QB_CE** | **34** | **0.309** | **0.976** | **-0.034** | **0.568** |
| **T1_QB_OE** | **C_QB_OE** | **34** | **0.245** | **0.545** | **-0.099** | **0.521** |
| **T1_QB_OE** | **C_QB_RT** | **34** | **0.474** | **9.977** | **0.150** | **0.684** |
| **T1_QB_OE** | **C_QB_RTV** | **34** | **0.320** | **1.092** | **-0.022** | **0.576** |
| **T1_QB_OE** | **T1_CPT_II_OE_Value** | **36** | **0.434** | **5.975** | **0.114** | **0.652** |
| **T1_QB_OE** | **T1_CPT_II_CE_Value** | **36** | **0.136** | **0.280** | **-0.195** | **0.429** |
| **T1_QB_OE** | **T1_CPT_II_Hit RT_Value** | **36** | **0.386** | **2.774** | **0.059** | **0.617** |
| **T1_QB_OE** | **T1_CPT_II_RTV_Value** | **36** | **0.455** | **8.765** | **0.139** | **0.666** |
| **T1_QB_OE** | **T2_CPT_II_OE_Value** | **34** | **0.186** | **0.363** | **-0.157** | **0.475** |
| **T1_QB_OE** | **T2_CPT_II_CE_Value** | **34** | **0.160** | **0.316** | **-0.181** | **0.455** |
| **T1_QB_OE** | **T2_CPT_II_Hit RT_Value** | **34** | **0.197** | **0.387** | **-0.146** | **0.484** |
| **T1_QB_OE** | **T2_CPT_II_RTV_Value** | **34** | **0.224** | **0.466** | **-0.119** | **0.505** |
| **T1_QB_OE** | **C_CPT_II_OE_Value** | **34** | **0.428** | **4.566** | **0.096** | **0.653** |
| **T1_QB_OE** | **C_CPT_II_CE_Value** | **34** | **-0.093** | **0.243** | **-0.402** | **0.243** |
| **T1_QB_OE** | **C_CPT_II_Hit RT_Value** | **34** | **0.375** | **2.112** | **0.037** | **0.616** |
| **T1_QB_OE** | **C_CPT_II_RTV_Value** | **34** | **0.448** | **6.339** | **0.120** | **0.667** |
| **T1_QB_RT** | **T1_QB_RTV** | **36** | **0.667** | **2678.890** | **0.411** | **0.806** |
| **T1_QB_RT** | **T2_QB_MicroEventX** | **34** | **-0.015** | **0.214** | **-0.338** | **0.311** |
| **T1_QB_RT** | **T2_QB_CE** | **34** | **-0.011** | **0.214** | **-0.334** | **0.315** |
| **T1_QB_RT** | **T2_QB_OE** | **34** | **0.399** | **2.942** | **0.063** | **0.632** |
| **T1_QB_RT** | **T2_QB_RT** | **34** | **0.779** | **279822.076** | **0.573** | **0.877** |
| **T1_QB_RT** | **T2_QB_RTV** | **34** | **0.469** | **9.081** | **0.144** | **0.680** |
| **T1_QB_RT** | **C_QB_MicroEventX** | **34** | **-0.115** | **0.261** | **-0.420** | **0.223** |
| **T1_QB_RT** | **C_QB_CE** | **34** | **0.208** | **0.417** | **-0.135** | **0.493** |
| **T1_QB_RT** | **C_QB_OE** | **34** | **0.122** | **0.268** | **-0.216** | **0.426** |
| **T1_QB_RT** | **C_QB_RT** | **34** | **0.391** | **2.635** | **0.055** | **0.627** |
| **T1_QB_RT** | **C_QB_RTV** | **34** | **0.286** | **0.776** | **-0.057** | **0.551** |
| **T1_QB_RT** | **T1_CPT_II_OE_Value** | **36** | **0.202** | **0.405** | **-0.132** | **0.481** |
| **T1_QB_RT** | **T1_CPT_II_CE_Value** | **36** | **0.016** | **0.208** | **-0.302** | **0.330** |
| **T1_QB_RT** | **T1_CPT_II_Hit RT_Value** | **36** | **0.534** | **46.582** | **0.234** | **0.720** |
| **T1_QB_RT** | **T1_CPT_II_RTV_Value** | **36** | **0.308** | **1.032** | **-0.025** | **0.561** |
| **T1_QB_RT** | **T2_CPT_II_OE_Value** | **34** | **0.031** | **0.217** | **-0.298** | **0.351** |
| **T1_QB_RT** | **T2_CPT_II_CE_Value** | **34** | **0.094** | **0.244** | **-0.242** | **0.403** |
| **T1_QB_RT** | **T2_CPT_II_Hit RT_Value** | **34** | **0.418** | **3.890** | **0.085** | **0.646** |
| **T1_QB_RT** | **T2_CPT_II_RTV_Value** | **34** | **0.314** | **1.024** | **-0.029** | **0.572** |
| **T1_QB_RT** | **C_CPT_II_OE_Value** | **34** | **0.225** | **0.469** | **-0.119** | **0.506** |
| **T1_QB_RT** | **C_CPT_II_CE_Value** | **34** | **-0.080** | **0.235** | **-0.392** | **0.254** |
| **T1_QB_RT** | **C_CPT_II_Hit RT_Value** | **34** | **0.293** | **0.825** | **-0.051** | **0.556** |
| **T1_QB_RT** | **C_CPT_II_RTV_Value** | **34** | **0.196** | **0.386** | **-0.147** | **0.483** |
| **T1_QB_RTV** | **T2_QB_MicroEventX** | **34** | **0.172** | **0.336** | **-0.170** | **0.465** |
| **T1_QB_RTV** | **T2_QB_CE** | **34** | **0.415** | **3.702** | **0.081** | **0.643** |
| **T1_QB_RTV** | **T2_QB_OE** | **34** | **0.655** | **1023.838** | **0.385** | **0.802** |
| **T1_QB_RTV** | **T2_QB_RT** | **34** | **0.486** | **12.483** | **0.164** | **0.692** |
| **T1_QB_RTV** | **T2_QB_RTV** | **34** | **0.733** | **24064.163** | **0.500** | **0.850** |
| **T1_QB_RTV** | **C_QB_MicroEventX** | **34** | **0.049** | **0.221** | **-0.282** | **0.366** |
| **T1_QB_RTV** | **C_QB_CE** | **34** | **0.200** | **0.396** | **-0.143** | **0.487** |
| **T1_QB_RTV** | **C_QB_OE** | **34** | **-0.069** | **0.229** | **-0.382** | **0.264** |
| **T1_QB_RTV** | **C_QB_RT** | **34** | **0.304** | **0.927** | **-0.039** | **0.565** |
| **T1_QB_RTV** | **C_QB_RTV** | **34** | **0.397** | **2.844** | **0.061** | **0.631** |
| **T1_QB_RTV** | **T1_CPT_II_OE_Value** | **36** | **0.592** | **212.884** | **0.308** | **0.758** |
| **T1_QB_RTV** | **T1_CPT_II_CE_Value** | **36** | **0.322** | **1.214** | **-0.009** | **0.572** |
| **T1_QB_RTV** | **T1_CPT_II_Hit RT_Value** | **36** | **0.582** | **159.945** | **0.295** | **0.751** |
| **T1_QB_RTV** | **T1_CPT_II_RTV_Value** | **36** | **0.664** | **2377.032** | **0.407** | **0.804** |
| **T1_QB_RTV** | **T2_CPT_II_OE_Value** | **34** | **0.219** | **0.448** | **-0.125** | **0.501** |
| **T1_QB_RTV** | **T2_CPT_II_CE_Value** | **34** | **0.242** | **0.530** | **-0.102** | **0.518** |
| **T1_QB_RTV** | **T2_CPT_II_Hit RT_Value** | **34** | **0.372** | **2.028** | **0.033** | **0.613** |
| **T1_QB_RTV** | **T2_CPT_II_RTV_Value** | **34** | **0.414** | **3.635** | **0.080** | **0.643** |
| **T1_QB_RTV** | **C_CPT_II_OE_Value** | **34** | **0.558** | **59.956** | **0.254** | **0.740** |
| **T1_QB_RTV** | **C_CPT_II_CE_Value** | **34** | **0.048** | **0.221** | **-0.283** | **0.365** |
| **T1_QB_RTV** | **C_CPT_II_Hit RT_Value** | **34** | **0.420** | **3.982** | **0.086** | **0.647** |
| **T1_QB_RTV** | **C_CPT_II_RTV_Value** | **34** | **0.560** | **62.184** | **0.256** | **0.741** |
| **T2_QB_MicroEventX** | **T2_QB_CE** | **34** | **0.480** | **11.231** | **0.157** | **0.688** |
| **T2_QB_MicroEventX** | **T2_QB_OE** | **34** | **0.463** | **8.209** | **0.137** | **0.677** |
| **T2_QB_MicroEventX** | **T2_QB_RT** | **34** | **0.127** | **0.273** | **-0.212** | **0.430** |
| **T2_QB_MicroEventX** | **T2_QB_RTV** | **34** | **0.457** | **7.389** | **0.130** | **0.673** |
| **T2_QB_MicroEventX** | **C_QB_MicroEventX** | **34** | **-0.498** | **15.756** | **-0.700** | **-0.178** |
| **T2_QB_MicroEventX** | **C_QB_CE** | **34** | **-0.161** | **0.318** | **-0.456** | **0.180** |
| **T2_QB_MicroEventX** | **C_QB_OE** | **34** | **-0.510** | **20.281** | **-0.708** | **-0.193** |
| **T2_QB_MicroEventX** | **C_QB_RT** | **34** | **-0.210** | **0.423** | **-0.494** | **0.133** |
| **T2_QB_MicroEventX** | **C_QB_RTV** | **34** | **-0.378** | **2.208** | **-0.618** | **-0.040** |
| **T2_QB_MicroEventX** | **T1_CPT_II_OE_Value** | **34** | **0.198** | **0.392** | **-0.145** | **0.485** |
| **T2_QB_MicroEventX** | **T1_CPT_II_CE_Value** | **34** | **0.277** | **0.714** | **-0.067** | **0.545** |
| **T2_QB_MicroEventX** | **T1_CPT_II_Hit RT_Value** | **34** | **-0.031** | **0.217** | **-0.351** | **0.298** |
| **T2_QB_MicroEventX** | **T1_CPT_II_RTV_Value** | **34** | **0.174** | **0.341** | **-0.168** | **0.467** |
| **T2_QB_MicroEventX** | **T2_CPT_II_OE_Value** | **34** | **0.527** | **28.956** | **0.214** | **0.719** |
| **T2_QB_MicroEventX** | **T2_CPT_II_CE_Value** | **34** | **0.472** | **9.705** | **0.148** | **0.683** |
| **T2_QB_MicroEventX** | **T2_CPT_II_Hit RT_Value** | **34** | **0.212** | **0.428** | **-0.131** | **0.496** |
| **T2_QB_MicroEventX** | **T2_CPT_II_RTV_Value** | **34** | **0.459** | **7.605** | **0.132** | **0.674** |
| **T2_QB_MicroEventX** | **C_CPT_II_OE_Value** | **34** | **-0.169** | **0.330** | **-0.462** | **0.173** |
| **T2_QB_MicroEventX** | **C_CPT_II_CE_Value** | **34** | **-0.329** | **1.206** | **-0.583** | **0.013** |
| **T2_QB_MicroEventX** | **C_CPT_II_Hit RT_Value** | **34** | **-0.254** | **0.587** | **-0.528** | **0.090** |
| **T2_QB_MicroEventX** | **C_CPT_II_RTV_Value** | **34** | **-0.048** | **0.221** | **-0.365** | **0.283** |
| **T2_QB_CE** | **T2_QB_OE** | **34** | **0.630** | **443.185** | **0.349** | **0.786** |
| **T2_QB_CE** | **T2_QB_RT** | **34** | **0.010** | **0.214** | **-0.316** | **0.333** |
| **T2_QB_CE** | **T2_QB_RTV** | **34** | **0.527** | **29.201** | **0.214** | **0.720** |
| **T2_QB_CE** | **C_QB_MicroEventX** | **34** | **-0.247** | **0.554** | **-0.522** | **0.097** |
| **T2_QB_CE** | **C_QB_CE** | **34** | **-0.485** | **12.192** | **-0.691** | **-0.162** |
| **T2_QB_CE** | **C_QB_OE** | **34** | **-0.548** | **47.039** | **-0.733** | **-0.241** |
| **T2_QB_CE** | **C_QB_RT** | **34** | **-0.031** | **0.217** | **-0.351** | **0.297** |
| **T2_QB_CE** | **C_QB_RTV** | **34** | **-0.136** | **0.284** | **-0.437** | **0.203** |
| **T2_QB_CE** | **T1_CPT_II_OE_Value** | **34** | **0.383** | **2.336** | **0.045** | **0.621** |
| **T2_QB_CE** | **T1_CPT_II_CE_Value** | **34** | **0.418** | **3.882** | **0.085** | **0.646** |
| **T2_QB_CE** | **T1_CPT_II_Hit RT_Value** | **34** | **0.166** | **0.326** | **-0.175** | **0.460** |
| **T2_QB_CE** | **T1_CPT_II_RTV_Value** | **34** | **0.422** | **4.151** | **0.089** | **0.649** |
| **T2_QB_CE** | **T2_CPT_II_OE_Value** | **34** | **0.525** | **27.576** | **0.211** | **0.718** |
| **T2_QB_CE** | **T2_CPT_II_CE_Value** | **34** | **0.495** | **15.034** | **0.175** | **0.698** |
| **T2_QB_CE** | **T2_CPT_II_Hit RT_Value** | **34** | **0.153** | **0.306** | **-0.187** | **0.450** |
| **T2_QB_CE** | **T2_CPT_II_RTV_Value** | **34** | **0.633** | **485.536** | **0.353** | **0.788** |
| **T2_QB_CE** | **C_CPT_II_OE_Value** | **34** | **0.048** | **0.221** | **-0.283** | **0.365** |
| **T2_QB_CE** | **C_CPT_II_CE_Value** | **34** | **-0.203** | **0.403** | **-0.489** | **0.140** |
| **T2_QB_CE** | **C_CPT_II_Hit RT_Value** | **34** | **0.064** | **0.227** | **-0.269** | **0.378** |
| **T2_QB_CE** | **C_CPT_II_RTV_Value** | **34** | **0.139** | **0.287** | **-0.201** | **0.439** |
| **T2_QB_OE** | **T2_QB_RT** | **34** | **0.374** | **2.094** | **0.036** | **0.615** |
| **T2_QB_OE** | **T2_QB_RTV** | **34** | **0.724** | **16114.006** | **0.487** | **0.845** |
| **T2_QB_OE** | **C_QB_MicroEventX** | **34** | **-0.088** | **0.240** | **-0.398** | **0.247** |
| **T2_QB_OE** | **C_QB_CE** | **34** | **-0.016** | **0.214** | **-0.338** | **0.311** |
| **T2_QB_OE** | **C_QB_OE** | **34** | **-0.571** | **82.929** | **-0.748** | **-0.270** |
| **T2_QB_OE** | **C_QB_RT** | **34** | **0.063** | **0.227** | **-0.270** | **0.377** |
| **T2_QB_OE** | **C_QB_RTV** | **34** | **-0.069** | **0.229** | **-0.383** | **0.264** |
| **T2_QB_OE** | **T1_CPT_II_OE_Value** | **34** | **0.642** | **640.605** | **0.365** | **0.793** |
| **T2_QB_OE** | **T1_CPT_II_CE_Value** | **34** | **0.210** | **0.422** | **-0.134** | **0.494** |
| **T2_QB_OE** | **T1_CPT_II_Hit RT_Value** | **34** | **0.519** | **24.641** | **0.205** | **0.714** |
| **T2_QB_OE** | **T1_CPT_II_RTV_Value** | **34** | **0.662** | **1283.525** | **0.394** | **0.806** |
| **T2_QB_OE** | **T2_CPT_II_OE_Value** | **34** | **0.507** | **19.085** | **0.190** | **0.706** |
| **T2_QB_OE** | **T2_CPT_II_CE_Value** | **34** | **0.415** | **3.715** | **0.081** | **0.644** |
| **T2_QB_OE** | **T2_CPT_II_Hit RT_Value** | **34** | **0.390** | **2.577** | **0.053** | **0.626** |
| **T2_QB_OE** | **T2_CPT_II_RTV_Value** | **34** | **0.486** | **12.613** | **0.165** | **0.692** |
| **T2_QB_OE** | **C_CPT_II_OE_Value** | **34** | **0.364** | **1.818** | **0.024** | **0.607** |
| **T2_QB_OE** | **C_CPT_II_CE_Value** | **34** | **-0.326** | **1.171** | **-0.581** | **0.016** |
| **T2_QB_OE** | **C_CPT_II_Hit RT_Value** | **34** | **0.289** | **0.794** | **-0.055** | **0.553** |
| **T2_QB_OE** | **C_CPT_II_RTV_Value** | **34** | **0.488** | **12.990** | **0.166** | **0.693** |
| **T2_QB_RT** | **T2_QB_RTV** | **34** | **0.607** | **219.629** | **0.318** | **0.771** |
| **T2_QB_RT** | **C_QB_MicroEventX** | **34** | **-0.282** | **0.750** | **-0.549** | **0.061** |
| **T2_QB_RT** | **C_QB_CE** | **34** | **0.223** | **0.461** | **-0.121** | **0.504** |
| **T2_QB_RT** | **C_QB_OE** | **34** | **-0.168** | **0.329** | **-0.462** | **0.174** |
| **T2_QB_RT** | **C_QB_RT** | **34** | **-0.272** | **0.682** | **-0.541** | **0.072** |
| **T2_QB_RT** | **C_QB_RTV** | **34** | **-0.146** | **0.295** | **-0.444** | **0.195** |
| **T2_QB_RT** | **T1_CPT_II_OE_Value** | **34** | **0.192** | **0.377** | **-0.151** | **0.480** |
| **T2_QB_RT** | **T1_CPT_II_CE_Value** | **34** | **0.079** | **0.234** | **-0.256** | **0.390** |
| **T2_QB_RT** | **T1_CPT_II_Hit RT_Value** | **34** | **0.559** | **61.008** | **0.255** | **0.740** |
| **T2_QB_RT** | **T1_CPT_II_RTV_Value** | **34** | **0.266** | **0.648** | **-0.078** | **0.536** |
| **T2_QB_RT** | **T2_CPT_II_OE_Value** | **34** | **0.163** | **0.321** | **-0.178** | **0.458** |
| **T2_QB_RT** | **T2_CPT_II_CE_Value** | **34** | **0.006** | **0.213** | **-0.319** | **0.330** |
| **T2_QB_RT** | **T2_CPT_II_Hit RT_Value** | **34** | **0.685** | **3088.982** | **0.428** | **0.821** |
| **T2_QB_RT** | **T2_CPT_II_RTV_Value** | **34** | **0.375** | **2.100** | **0.036** | **0.615** |
| **T2_QB_RT** | **C_CPT_II_OE_Value** | **34** | **0.100** | **0.249** | **-0.236** | **0.408** |
| **T2_QB_RT** | **C_CPT_II_CE_Value** | **34** | **0.079** | **0.235** | **-0.255** | **0.391** |
| **T2_QB_RT** | **C_CPT_II_Hit RT_Value** | **34** | **0.043** | **0.219** | **-0.287** | **0.361** |
| **T2_QB_RT** | **C_CPT_II_RTV_Value** | **34** | **0.101** | **0.249** | **-0.236** | **0.408** |
| **T2_QB_RTV** | **C_QB_MicroEventX** | **34** | **-0.288** | **0.790** | **-0.553** | **0.056** |
| **T2_QB_RTV** | **C_QB_CE** | **34** | **0.075** | **0.233** | **-0.259** | **0.388** |
| **T2_QB_RTV** | **C_QB_OE** | **34** | **-0.395** | **2.757** | **-0.629** | **-0.058** |
| **T2_QB_RTV** | **C_QB_RT** | **34** | **-0.171** | **0.334** | **-0.464** | **0.171** |
| **T2_QB_RTV** | **C_QB_RTV** | **34** | **-0.333** | **1.268** | **-0.586** | **0.008** |
| **T2_QB_RTV** | **T1_CPT_II_OE_Value** | **34** | **0.559** | **61.266** | **0.255** | **0.741** |
| **T2_QB_RTV** | **T1_CPT_II_CE_Value** | **34** | **0.376** | **2.133** | **0.038** | **0.616** |
| **T2_QB_RTV** | **T1_CPT_II_Hit RT_Value** | **34** | **0.502** | **17.139** | **0.183** | **0.703** |
| **T2_QB_RTV** | **T1_CPT_II_RTV_Value** | **34** | **0.592** | **145.416** | **0.298** | **0.762** |
| **T2_QB_RTV** | **T2_CPT_II_OE_Value** | **34** | **0.521** | **25.439** | **0.207** | **0.715** |
| **T2_QB_RTV** | **T2_CPT_II_CE_Value** | **34** | **0.287** | **0.785** | **-0.056** | **0.552** |
| **T2_QB_RTV** | **T2_CPT_II_Hit RT_Value** | **34** | **0.588** | **130.786** | **0.293** | **0.760** |
| **T2_QB_RTV** | **T2_CPT_II_RTV_Value** | **34** | **0.612** | **256.984** | **0.325** | **0.775** |
| **T2_QB_RTV** | **C_CPT_II_OE_Value** | **34** | **0.257** | **0.599** | **-0.087** | **0.530** |
| **T2_QB_RTV** | **C_CPT_II_CE_Value** | **34** | **0.031** | **0.217** | **-0.298** | **0.351** |
| **T2_QB_RTV** | **C_CPT_II_Hit RT_Value** | **34** | **0.066** | **0.228** | **-0.267** | **0.380** |
| **T2_QB_RTV** | **C_CPT_II_RTV_Value** | **34** | **0.342** | **1.402** | **0.001** | **0.592** |
| **C_QB_MicroEventX** | **C_QB_CE** | **34** | **0.172** | **0.336** | **-0.170** | **0.465** |
| **C_QB_MicroEventX** | **C_QB_OE** | **34** | **0.194** | **0.382** | **-0.149** | **0.482** |
| **C_QB_MicroEventX** | **C_QB_RT** | **34** | **0.238** | **0.516** | **-0.106** | **0.516** |
| **C_QB_MicroEventX** | **C_QB_RTV** | **34** | **0.456** | **7.245** | **0.129** | **0.672** |
| **C_QB_MicroEventX** | **T1_CPT_II_OE_Value** | **34** | **0.119** | **0.265** | **-0.219** | **0.423** |
| **C_QB_MicroEventX** | **T1_CPT_II_CE_Value** | **34** | **0.040** | **0.219** | **-0.290** | **0.358** |
| **C_QB_MicroEventX** | **T1_CPT_II_Hit RT_Value** | **34** | **0.032** | **0.217** | **-0.297** | **0.352** |
| **C_QB_MicroEventX** | **T1_CPT_II_RTV_Value** | **34** | **0.085** | **0.238** | **-0.250** | **0.395** |
| **C_QB_MicroEventX** | **T2_CPT_II_OE_Value** | **34** | **-0.199** | **0.393** | **-0.486** | **0.144** |
| **C_QB_MicroEventX** | **T2_CPT_II_CE_Value** | **34** | **-0.134** | **0.281** | **-0.435** | **0.206** |
| **C_QB_MicroEventX** | **T2_CPT_II_Hit RT_Value** | **34** | **-0.115** | **0.261** | **-0.420** | **0.223** |
| **C_QB_MicroEventX** | **T2_CPT_II_RTV_Value** | **34** | **-0.382** | **2.302** | **-0.620** | **-0.044** |
| **C_QB_MicroEventX** | **C_CPT_II_OE_Value** | **34** | **0.290** | **0.804** | **-0.054** | **0.554** |
| **C_QB_MicroEventX** | **C_CPT_II_CE_Value** | **34** | **0.225** | **0.469** | **-0.119** | **0.506** |
| **C_QB_MicroEventX** | **C_CPT_II_Hit RT_Value** | **34** | **0.157** | **0.312** | **-0.184** | **0.453** |
| **C_QB_MicroEventX** | **C_CPT_II_RTV_Value** | **34** | **0.300** | **0.885** | **-0.044** | **0.561** |
| **C_QB_CE** | **C_QB_OE** | **34** | **0.357** | **1.678** | **0.017** | **0.603** |
| **C_QB_CE** | **C_QB_RT** | **34** | **-0.007** | **0.214** | **-0.331** | **0.318** |
| **C_QB_CE** | **C_QB_RTV** | **34** | **0.176** | **0.343** | **-0.166** | **0.468** |
| **C_QB_CE** | **T1_CPT_II_OE_Value** | **34** | **0.159** | **0.314** | **-0.183** | **0.454** |
| **C_QB_CE** | **T1_CPT_II_CE_Value** | **34** | **-0.090** | **0.241** | **-0.399** | **0.246** |
| **C_QB_CE** | **T1_CPT_II_Hit RT_Value** | **34** | **0.273** | **0.686** | **-0.071** | **0.541** |
| **C_QB_CE** | **T1_CPT_II_RTV_Value** | **34** | **0.150** | **0.301** | **-0.190** | **0.448** |
| **C_QB_CE** | **T2_CPT_II_OE_Value** | **34** | **-0.238** | **0.516** | **-0.515** | **0.106** |
| **C_QB_CE** | **T2_CPT_II_CE_Value** | **34** | **-0.317** | **1.056** | **-0.574** | **0.026** |
| **C_QB_CE** | **T2_CPT_II_Hit RT_Value** | **34** | **0.271** | **0.676** | **-0.073** | **0.540** |
| **C_QB_CE** | **T2_CPT_II_RTV_Value** | **34** | **-0.266** | **0.648** | **-0.536** | **0.078** |
| **C_QB_CE** | **C_CPT_II_OE_Value** | **34** | **0.366** | **1.879** | **0.027** | **0.609** |
| **C_QB_CE** | **C_CPT_II_CE_Value** | **34** | **0.328** | **1.188** | **-0.014** | **0.582** |
| **C_QB_CE** | **C_CPT_II_Hit RT_Value** | **34** | **0.085** | **0.238** | **-0.250** | **0.395** |
| **C_QB_CE** | **C_CPT_II_RTV_Value** | **34** | **0.312** | **1.000** | **-0.031** | **0.570** |
| **C_QB_OE** | **C_QB_RT** | **34** | **0.434** | **5.036** | **0.103** | **0.657** |
| **C_QB_OE** | **C_QB_RTV** | **34** | **0.437** | **5.239** | **0.106** | **0.659** |
| **C_QB_OE** | **T1_CPT_II_OE_Value** | **34** | **-0.294** | **0.833** | **-0.557** | **0.050** |
| **C_QB_OE** | **T1_CPT_II_CE_Value** | **34** | **-0.150** | **0.301** | **-0.448** | **0.191** |
| **C_QB_OE** | **T1_CPT_II_Hit RT_Value** | **34** | **-0.192** | **0.375** | **-0.480** | **0.151** |
| **C_QB_OE** | **T1_CPT_II_RTV_Value** | **34** | **-0.303** | **0.915** | **-0.564** | **0.040** |
| **C_QB_OE** | **T2_CPT_II_OE_Value** | **34** | **-0.450** | **6.467** | **-0.667** | **-0.121** |
| **C_QB_OE** | **T2_CPT_II_CE_Value** | **34** | **-0.359** | **1.722** | **-0.604** | **-0.019** |
| **C_QB_OE** | **T2_CPT_II_Hit RT_Value** | **34** | **-0.287** | **0.781** | **-0.552** | **0.057** |
| **C_QB_OE** | **T2_CPT_II_RTV_Value** | **34** | **-0.381** | **2.274** | **-0.619** | **-0.043** |
| **C_QB_OE** | **C_CPT_II_OE_Value** | **34** | **-0.001** | **0.213** | **-0.325** | **0.324** |
| **C_QB_OE** | **C_CPT_II_CE_Value** | **34** | **0.318** | **1.066** | **-0.025** | **0.574** |
| **C_QB_OE** | **C_CPT_II_Hit RT_Value** | **34** | **0.037** | **0.218** | **-0.292** | **0.356** |
| **C_QB_OE** | **C_CPT_II_RTV_Value** | **34** | **-0.139** | **0.287** | **-0.439** | **0.201** |
| **C_QB_RT** | **C_QB_RTV** | **34** | **0.653** | **932.922** | **0.381** | **0.800** |
| **C_QB_RT** | **T1_CPT_II_OE_Value** | **34** | **0.045** | **0.220** | **-0.286** | **0.363** |
| **C_QB_RT** | **T1_CPT_II_CE_Value** | **34** | **-0.052** | **0.222** | **-0.368** | **0.280** |
| **C_QB_RT** | **T1_CPT_II_Hit RT_Value** | **34** | **0.015** | **0.214** | **-0.312** | **0.337** |
| **C_QB_RT** | **T1_CPT_II_RTV_Value** | **34** | **0.103** | **0.251** | **-0.234** | **0.410** |
| **C_QB_RT** | **T2_CPT_II_OE_Value** | **34** | **-0.192** | **0.377** | **-0.480** | **0.151** |
| **C_QB_RT** | **T2_CPT_II_CE_Value** | **34** | **0.135** | **0.282** | **-0.205** | **0.436** |
| **C_QB_RT** | **T2_CPT_II_Hit RT_Value** | **34** | **-0.364** | **1.840** | **-0.608** | **-0.025** |
| **C_QB_RT** | **T2_CPT_II_RTV_Value** | **34** | **-0.068** | **0.229** | **-0.382** | **0.265** |
| **C_QB_RT** | **C_CPT_II_OE_Value** | **34** | **0.199** | **0.392** | **-0.145** | **0.485** |
| **C_QB_RT** | **C_CPT_II_CE_Value** | **34** | **-0.240** | **0.522** | **-0.517** | **0.104** |
| **C_QB_RT** | **C_CPT_II_Hit RT_Value** | **34** | **0.386** | **2.435** | **0.048** | **0.623** |
| **C_QB_RT** | **C_CPT_II_RTV_Value** | **34** | **0.153** | **0.305** | **-0.188** | **0.450** |
| **C_QB_RTV** | **T1_CPT_II_OE_Value** | **34** | **0.106** | **0.253** | **-0.231** | **0.412** |
| **C_QB_RTV** | **T1_CPT_II_CE_Value** | **34** | **-0.042** | **0.219** | **-0.360** | **0.288** |
| **C_QB_RTV** | **T1_CPT_II_Hit RT_Value** | **34** | **0.162** | **0.319** | **-0.179** | **0.457** |
| **C_QB_RTV** | **T1_CPT_II_RTV_Value** | **34** | **0.158** | **0.313** | **-0.183** | **0.454** |
| **C_QB_RTV** | **T2_CPT_II_OE_Value** | **34** | **-0.399** | **2.953** | **-0.633** | **-0.064** |
| **C_QB_RTV** | **T2_CPT_II_CE_Value** | **34** | **-0.053** | **0.223** | **-0.369** | **0.279** |
| **C_QB_RTV** | **T2_CPT_II_Hit RT_Value** | **34** | **-0.278** | **0.723** | **-0.546** | **0.065** |
| **C_QB_RTV** | **T2_CPT_II_RTV_Value** | **34** | **-0.253** | **0.580** | **-0.527** | **0.091** |
| **C_QB_RTV** | **C_CPT_II_OE_Value** | **34** | **0.427** | **4.470** | **0.095** | **0.652** |
| **C_QB_RTV** | **C_CPT_II_CE_Value** | **34** | **0.025** | **0.215** | **-0.303** | **0.345** |
| **C_QB_RTV** | **C_CPT_II_Hit RT_Value** | **34** | **0.492** | **14.129** | **0.172** | **0.696** |
| **C_QB_RTV** | **C_CPT_II_RTV_Value** | **34** | **0.314** | **1.024** | **-0.029** | **0.572** |
| **T1_CPT_II_OE_Value** | **T1_CPT_II_CE_Value** | **36** | **0.046** | **0.215** | **-0.276** | **0.355** |
| **T1_CPT_II_OE_Value** | **T1_CPT_II_Hit RT_Value** | **36** | **0.774** | **511696.788** | **0.574** | **0.872** |
| **T1_CPT_II_OE_Value** | **T1_CPT_II_RTV_Value** | **36** | **0.938** | **1.130×10^+14^** | **0.867** | **0.967** |
| **T1_CPT_II_OE_Value** | **T2_CPT_II_OE_Value** | **34** | **0.530** | **31.103** | **0.218** | **0.721** |
| **T1_CPT_II_OE_Value** | **T2_CPT_II_CE_Value** | **34** | **0.041** | **0.219** | **-0.289** | **0.359** |
| **T1_CPT_II_OE_Value** | **T2_CPT_II_Hit RT_Value** | **34** | **0.408** | **3.367** | **0.074** | **0.639** |
| **T1_CPT_II_OE_Value** | **T2_CPT_II_RTV_Value** | **34** | **0.387** | **2.488** | **0.050** | **0.624** |
| **T1_CPT_II_OE_Value** | **C_CPT_II_OE_Value** | **34** | **0.765** | **122690.425** | **0.550** | **0.869** |
| **T1_CPT_II_OE_Value** | **C_CPT_II_CE_Value** | **34** | **0.009** | **0.214** | **-0.317** | **0.332** |
| **T1_CPT_II_OE_Value** | **C_CPT_II_Hit RT_Value** | **34** | **0.602** | **193.728** | **0.312** | **0.769** |
| **T1_CPT_II_OE_Value** | **C_CPT_II_RTV_Value** | **34** | **0.852** | **6.392×10^+7^** | **0.699** | **0.920** |
| **T1_CPT_II_CE_Value** | **T1_CPT_II_Hit RT_Value** | **36** | **-0.221** | **0.465** | **-0.496** | **0.113** |
| **T1_CPT_II_CE_Value** | **T1_CPT_II_RTV_Value** | **36** | **0.040** | **0.213** | **-0.281** | **0.350** |
| **T1_CPT_II_CE_Value** | **T2_CPT_II_OE_Value** | **34** | **0.093** | **0.243** | **-0.243** | **0.402** |
| **T1_CPT_II_CE_Value** | **T2_CPT_II_CE_Value** | **34** | **0.686** | **3169.254** | **0.429** | **0.821** |
| **T1_CPT_II_CE_Value** | **T2_CPT_II_Hit RT_Value** | **34** | **-0.045** | **0.220** | **-0.363** | **0.285** |
| **T1_CPT_II_CE_Value** | **T2_CPT_II_RTV_Value** | **34** | **0.362** | **1.777** | **0.022** | **0.606** |
| **T1_CPT_II_CE_Value** | **C_CPT_II_OE_Value** | **34** | **-0.002** | **0.213** | **-0.326** | **0.323** |
| **T1_CPT_II_CE_Value** | **C_CPT_II_CE_Value** | **34** | **0.188** | **0.368** | **-0.155** | **0.477** |
| **T1_CPT_II_CE_Value** | **C_CPT_II_Hit RT_Value** | **34** | **-0.224** | **0.465** | **-0.505** | **0.120** |
| **T1_CPT_II_CE_Value** | **C_CPT_II_RTV_Value** | **34** | **-0.139** | **0.287** | **-0.439** | **0.201** |
| **T1_CPT_II_Hit RT_Value** | **T1_CPT_II_RTV_Value** | **36** | **0.840** | **7.270×10^+7^** | **0.685** | **0.911** |
| **T1_CPT_II_Hit RT_Value** | **T2_CPT_II_OE_Value** | **34** | **0.277** | **0.713** | **-0.067** | **0.544** |
| **T1_CPT_II_Hit RT_Value** | **T2_CPT_II_CE_Value** | **34** | **-0.187** | **0.366** | **-0.477** | **0.155** |
| **T1_CPT_II_Hit RT_Value** | **T2_CPT_II_Hit RT_Value** | **34** | **0.656** | **1030.394** | **0.385** | **0.802** |
| **T1_CPT_II_Hit RT_Value** | **T2_CPT_II_RTV_Value** | **34** | **0.354** | **1.612** | **0.014** | **0.600** |
| **T1_CPT_II_Hit RT_Value** | **C_CPT_II_OE_Value** | **34** | **0.692** | **4059.343** | **0.438** | **0.825** |
| **T1_CPT_II_Hit RT_Value** | **C_CPT_II_CE_Value** | **34** | **0.023** | **0.215** | **-0.304** | **0.344** |
| **T1_CPT_II_Hit RT_Value** | **C_CPT_II_Hit RT_Value** | **34** | **0.651** | **883.078** | **0.379** | **0.799** |
| **T1_CPT_II_Hit RT_Value** | **C_CPT_II_RTV_Value** | **34** | **0.761** | **101461.147** | **0.544** | **0.867** |
| **T1_CPT_II_RTV_Value** | **T2_CPT_II_OE_Value** | **34** | **0.464** | **8.293** | **0.138** | **0.677** |
| **T1_CPT_II_RTV_Value** | **T2_CPT_II_CE_Value** | **34** | **0.066** | **0.228** | **-0.267** | **0.380** |
| **T1_CPT_II_RTV_Value** | **T2_CPT_II_Hit RT_Value** | **34** | **0.474** | **10.019** | **0.150** | **0.684** |
| **T1_CPT_II_RTV_Value** | **T2_CPT_II_RTV_Value** | **34** | **0.465** | **8.463** | **0.139** | **0.678** |
| **T1_CPT_II_RTV_Value** | **C_CPT_II_OE_Value** | **34** | **0.742** | **37098.365** | **0.514** | **0.855** |
| **T1_CPT_II_RTV_Value** | **C_CPT_II_CE_Value** | **34** | **-0.036** | **0.218** | **-0.355** | **0.294** |
| **T1_CPT_II_RTV_Value** | **C_CPT_II_Hit RT_Value** | **34** | **0.625** | **382.972** | **0.343** | **0.783** |
| **T1_CPT_II_RTV_Value** | **C_CPT_II_RTV_Value** | **34** | **0.881** | **1.388×10^+9^** | **0.753** | **0.936** |
| **T2_CPT_II_OE_Value** | **T2_CPT_II_CE_Value** | **34** | **0.181** | **0.353** | **-0.162** | **0.472** |
| **T2_CPT_II_OE_Value** | **T2_CPT_II_Hit RT_Value** | **34** | **0.481** | **11.334** | **0.158** | **0.689** |
| **T2_CPT_II_OE_Value** | **T2_CPT_II_RTV_Value** | **34** | **0.664** | **1366.211** | **0.396** | **0.807** |
| **T2_CPT_II_OE_Value** | **C_CPT_II_OE_Value** | **34** | **-0.141** | **0.289** | **-0.441** | **0.199** |
| **T2_CPT_II_OE_Value** | **C_CPT_II_CE_Value** | **34** | **-0.141** | **0.289** | **-0.440** | **0.199** |
| **T2_CPT_II_OE_Value** | **C_CPT_II_Hit RT_Value** | **34** | **-0.121** | **0.266** | **-0.424** | **0.218** |
| **T2_CPT_II_OE_Value** | **C_CPT_II_RTV_Value** | **34** | **0.170** | **0.332** | **-0.172** | **0.463** |
| **T2_CPT_II_CE_Value** | **T2_CPT_II_Hit RT_Value** | **34** | **-0.256** | **0.595** | **-0.529** | **0.088** |
| **T2_CPT_II_CE_Value** | **T2_CPT_II_RTV_Value** | **34** | **0.372** | **2.037** | **0.034** | **0.614** |
| **T2_CPT_II_CE_Value** | **C_CPT_II_OE_Value** | **34** | **-0.089** | **0.241** | **-0.399** | **0.246** |
| **T2_CPT_II_CE_Value** | **C_CPT_II_CE_Value** | **34** | **-0.585** | **120.902** | **-0.758** | **-0.289** |
| **T2_CPT_II_CE_Value** | **C_CPT_II_Hit RT_Value** | **34** | **0.012** | **0.214** | **-0.314** | **0.335** |
| **T2_CPT_II_CE_Value** | **C_CPT_II_RTV_Value** | **34** | **-0.124** | **0.270** | **-0.427** | **0.215** |
| **T2_CPT_II_Hit RT_Value** | **T2_CPT_II_RTV_Value** | **34** | **0.487** | **12.685** | **0.165** | **0.693** |
| **T2_CPT_II_Hit RT_Value** | **C_CPT_II_OE_Value** | **34** | **0.111** | **0.258** | **-0.226** | **0.417** |
| **T2_CPT_II_Hit RT_Value** | **C_CPT_II_CE_Value** | **34** | **0.295** | **0.845** | **-0.048** | **0.558** |
| **T2_CPT_II_Hit RT_Value** | **C_CPT_II_Hit RT_Value** | **34** | **-0.146** | **0.296** | **-0.445** | **0.194** |
| **T2_CPT_II_Hit RT_Value** | **C_CPT_II_RTV_Value** | **34** | **0.276** | **0.706** | **-0.068** | **0.544** |
| **T2_CPT_II_RTV_Value** | **C_CPT_II_OE_Value** | **34** | **-0.052** | **0.222** | **-0.368** | **0.279** |
| **T2_CPT_II_RTV_Value** | **C_CPT_II_CE_Value** | **34** | **-0.100** | **0.248** | **-0.407** | **0.237** |
| **T2_CPT_II_RTV_Value** | **C_CPT_II_Hit RT_Value** | **34** | **-0.026** | **0.216** | **-0.346** | **0.302** |
| **T2_CPT_II_RTV_Value** | **C_CPT_II_RTV_Value** | **34** | **-0.009** | **0.214** | **-0.332** | **0.317** |
| **C_CPT_II_OE_Value** | **C_CPT_II_CE_Value** | **34** | **0.118** | **0.264** | **-0.220** | **0.422** |
| **C_CPT_II_OE_Value** | **C_CPT_II_Hit RT_Value** | **34** | **0.795** | **753908.929** | **0.599** | **0.887** |
| **C_CPT_II_OE_Value** | **C_CPT_II_RTV_Value** | **34** | **0.866** | **2.481×10^+8^** | **0.724** | **0.928** |
| **C_CPT_II_CE_Value** | **C_CPT_II_Hit RT_Value** | **34** | **-0.266** | **0.646** | **-0.536** | **0.078** |
| **C_CPT_II_CE_Value** | **C_CPT_II_RTV_Value** | **34** | **0.013** | **0.214** | **-0.314** | **0.335** |
| **C_CPT_II_Hit RT_Value** | **C_CPT_II_RTV_Value** | **34** | **0.720** | **13166.663** | **0.480** | **0.842** |
| *Note.* BF₁₀ quantifies evidence for H₁ (correlation) over H₀ (no correlation). BF₁₀ < 1 suggests support for H₀. BF₁₀ > 3-10 moderate evidence for correlation. BF₁₀ > 10 -30 strong evidence for correlation. BF₁₀ > 100 extreme evidence for correlation.  BF: Bayes Factor, CE: commission errors, CI: Credible Interval, C: Change, CPT: Continuous Performance Tests, OE: omission errors, QB: QbCheck, *r*: Correlation Coefficient, RT: reaction time, RTV: reaction time variability, T1: baseline, T2: follow up, | | | | | | |
